# Transcriptome analysis of follicles reveals the importance of autophagy and hormones in regulating broodiness of Zhedong white goose

**DOI:** 10.1038/srep36877

**Published:** 2016-11-11

**Authors:** Jing Yu, Yaping Lou, Ayong Zhao

**Affiliations:** 1 College of Animal Science and Technology, Zhejiang Agriculture and Forestry University, 88 Huanbei Road, Lin’an 311300, China

## Abstract

Broodiness, a maternal behavior and instinct for natural breeding in poultry, inhibits egg production and affects the poultry industry. Phenotypic and physiological factors influencing broodiness in poultry have been extensively studied, but the molecular regulation mechanism of broodiness remains unclear. Effective research strategies focusing on broodiness are hindered by limited understanding of goose developmental biology. Here we established the transcriptomes of goose follicles at egg-laying and broody stages by Illumina HiSeq platform and compared the sequenced transcriptomes of three types of follicles (small white, large white and small yellow). It was found that there were 92 up-regulated and 84 down-regulated transcription factors and 101 up-regulated and 51 down-regulated hormone-related genes. Many of these genes code for proteins involved in hormone response, follicular development, autophagy, and oxidation. Moreover, the contents of progesterone and estradiol in follicles were altered, and the autophagy levels of follicles were enhanced during the broody stage. These results suggest that hormone- and autophagy-signaling pathways are critical for controlling broodiness in the goose. We demonstrated that transcriptome analysis of egg-laying and broody Zhedong white goose follicles provided novel insights into broodiness in birds.

The Zhedong white goose, characterized by strict seasonality, a high tendency to broodiness and incubation, and a low rate of egg laying, is one of the classical seasonal reproductive animals in China^1^. They have 3 or 4 laying cycles (Laying-Broodiness-Recovery) per year, and their laying periods are from October to April of the following year in Zhejiang province, China. The stable laying-broodiness cycles and easily identifiable characteristics of broodiness make this goose species an ideal model to study broodiness.

Broodiness, a common habit of most domestic fowls, reduces the egg production and is a major restricting element to industrial-scale production^2^. The number of eggs laid by a bird is determined by the number of follicles destined for ovulation and the capacity of the oviduct to transform the ova into a hard-shelled egg. Therefore, poultry laying performance is closely related to the development of follicles and the establishment of the follicular hierarchy^3^,^4^,^5^. However, large yellow follicles (LYF) and small yellow follicles (SYF) are almost absent on the ovary during broodiness, but the number of large white follicles (LWF) has no significant variation^6^. Therefore, the transition from LWF to SYF might be the key point to control the egg production in the goose. Interestingly, we found that the autophagy of LWF and SYF appeared stronger in the broody stage than in the egg-laying stage, which is considered the main reason for broodiness in geese^7^. However, it is necessary to explain the relationships between broodiness and the establishment of follicle grades.

Broodiness is associated with hereditary and other factors such as environment, nutrition, hormones, and so on. It is believed that the altered levels of reproductive endocrine hormones, including gonadotrophin (GnRH), prolactin (PRL), luteinizing hormone (LH), progesterone (P4), and estradiol (E2), are the major factors inducing the occurrence of broodiness^7^,^8^,^9^,^10^,^11^,^12^. The elevated levels of PRL, following the decrease of P4 and E2, induced the occurrence of broodiness^8^,^9^. Interestingly, in avian species, the granulosa cells only produce progesterone, and the theca folliculi produces testosterone or estradiol^13^. In female canaries and hens, progesterone dropped significantly at the onset of incubation^14^,^15^. Meanwhile, before incubation, the concentration of estradiol reduced and was maintained at low levels in the Chabo hen^16^. GnRH, synthesized and secreted by the hypothalamus, affects the ovarian activity controlling egg laying^17^.

Further studies showed that many other factors, including autophagy, homeostasis and ambient temperature, are associated with broodiness. An elevated ambient temperature accelerated the broody process in turkeys by increasing the level of PRL^10^. Our previous study indicated that the broodiness was associated with autophagy^7^. During the broody stage, the level of autophagy, identified by scanning electron microscope and the ratio of LC3-II to LC3-I, was elevated, and the homeostasis of follicles was impaired in goose follicles^7^. Also, many autophagy-related genes and miRNAs altered their expression in broody follicles tested by qPCR and miRNA transcriptome^7^,^18^. However, the mechanism of the occurrence of broodiness is not clear.

RNA-Seq, a next-generation sequencing technology, is a powerful tool to uncover transcriptional profiles for gene expression analysis to predict the possible molecular mechanism. RNA-Seq data are sufficient for gene model prediction, identification of novel transcripts, and measurement of transcript expression^19^,^20^. Moreover, RNA-Seq technology is much more sensitive and efficient for comparing gene expression profiles^20^. The successful sequencing of the goose genome opened a prelude to more detailed study of geese^21^. To date, RNA-Seq has been applied in geese, mainly based on the ovarian^22^,^23^, spleen^24^, exocrine^25^, hepatic^26^, follicles^27^, and mixed tissues^2^. Identified genes are responsible for reproductive biology, development and metabolism processes, and laying performance.

The genetic mechanism behind broody behavior has been widely studied for its potential effect on egg production. This mechanism of broodiness was considered to be controlled by a set of hormone-related genes of the ovarian tissues, including *PRL* and *PRLR* (prolactin receptor), and autophagy-related genes, including *p53, Beclin1, Atg12, Atg9*, and *Caspase3* in previous studies. However, based on our previous study, we speculated that their different expressions in follicles might be the cause inducing broodiness^7^. To deeply explore the mechanism of broodiness occurring in geese, we sampled the SWF, LWF, and SYF during egg-laying and broody stages, and analyzed the whole transcriptomic data. It was revealed that certain genes involved in pathways for reproduction regulation, such as hormone biosynthesis, phagosome, pathway in cancer, focal adhesion, and regulation of actin cytoskeleton, were differentially regulated.

## Results

### Transcriptome sequencing and quality control

To obtain a global view of the Zhedong white geese follicular transcriptome and identify the genes involved in the regulation of broodiness, cDNA libraries from the different grade follicles of broody and egg-laying geese were constructed and sequenced. Totals of 30,417,008 to 100,673,900 raw reads were generated in each library, and the valid data were 29,846,346 to 99,189,858 reads, with the valid ratios being more than 96% for 18 libraries. The data qualities were good, with Q20 (base sequencing error probability <1%) >98% and Q30 (base sequencing error probability <0.1%) >82% in each library (Table 1).

To determine the precision of estimated FPKMs at the current sequencing depth, an FPKM saturation analysis was performed. With an increasing number of reads, the number of detected genes increases as well. Moreover, we found the sample correlations were more than 0.8 (Table S1), suggesting that the results are reliable and reasonable. Furthermore, the evenly distributed reads in every position of the genes indicate that the randomness of the breaking of these 18 samples was good.

### Reads mapping to the reference genome dataset

**Figure 1:**
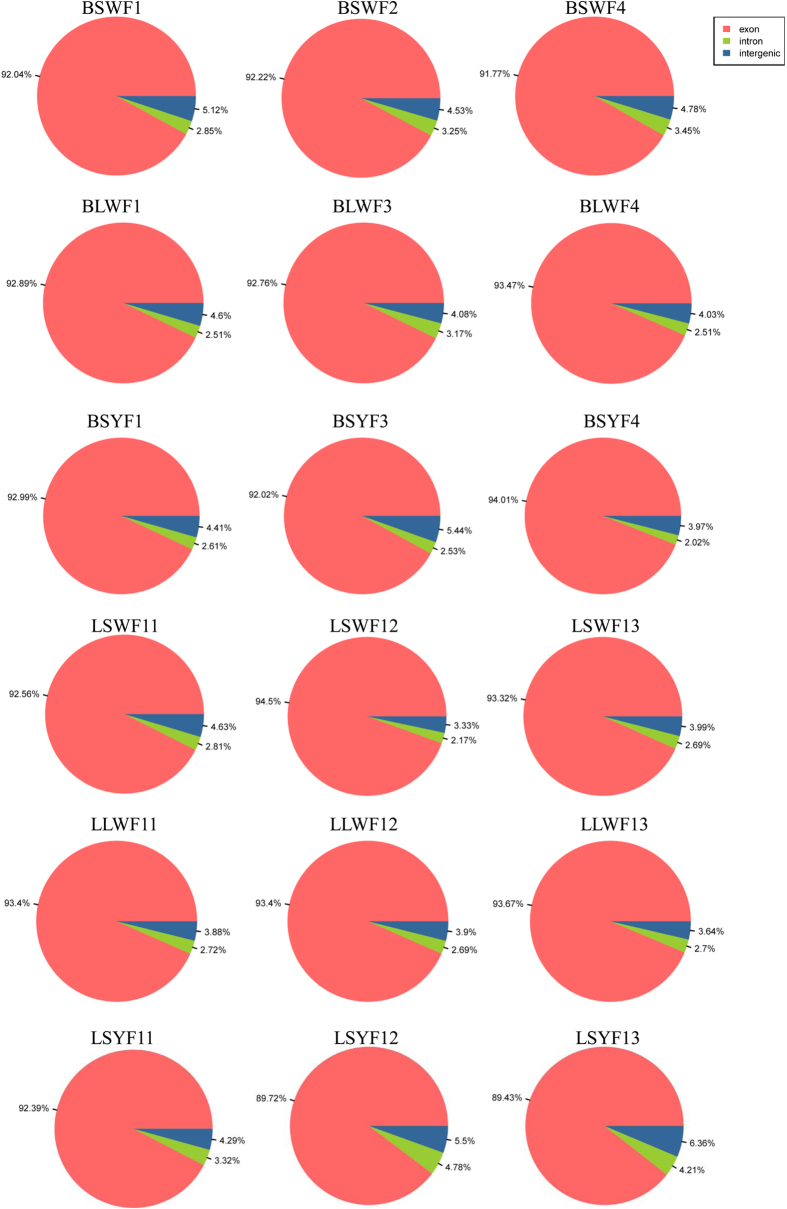
Distribution statistics of reads mapped to reference genes.

To identify the genes corresponding to clean reads in each library, the clean reads were mapped to the reference genes expressed in the goose genome. The mapping results show that 58.6% to 71.5% of the reads from each library were perfectly matched to the reference genome, whereas in unique mapped reads, 3.12% (371,746) to 3.45% (432,179), and in multi mapped reads, 3.12% (371,746) to 3.45% (432,179) were matched (Table S2). Gene expression of each sample showed a normal distribution (Figure S1), suggesting that the gene expression of three biological replicates had the consistent trend. Most importantly, 90% of the reads from each library were perfectly matched to the reference exons (Fig. 1).

Gene coverage was calculated as the absolute depth of read coverage across the genes from each of the broody and egg-laying goose follicles libraries. 0~1 of the absolute depth of the samples had coverage between 51~59%, and >30 of the absolute depth of the samples had coverage between 12~25% (Table 2). The results show that the sequencing quality complied with the requirements of further analyses.

### Analysis of differential expression genes (DEGs)

**Figure 2:**
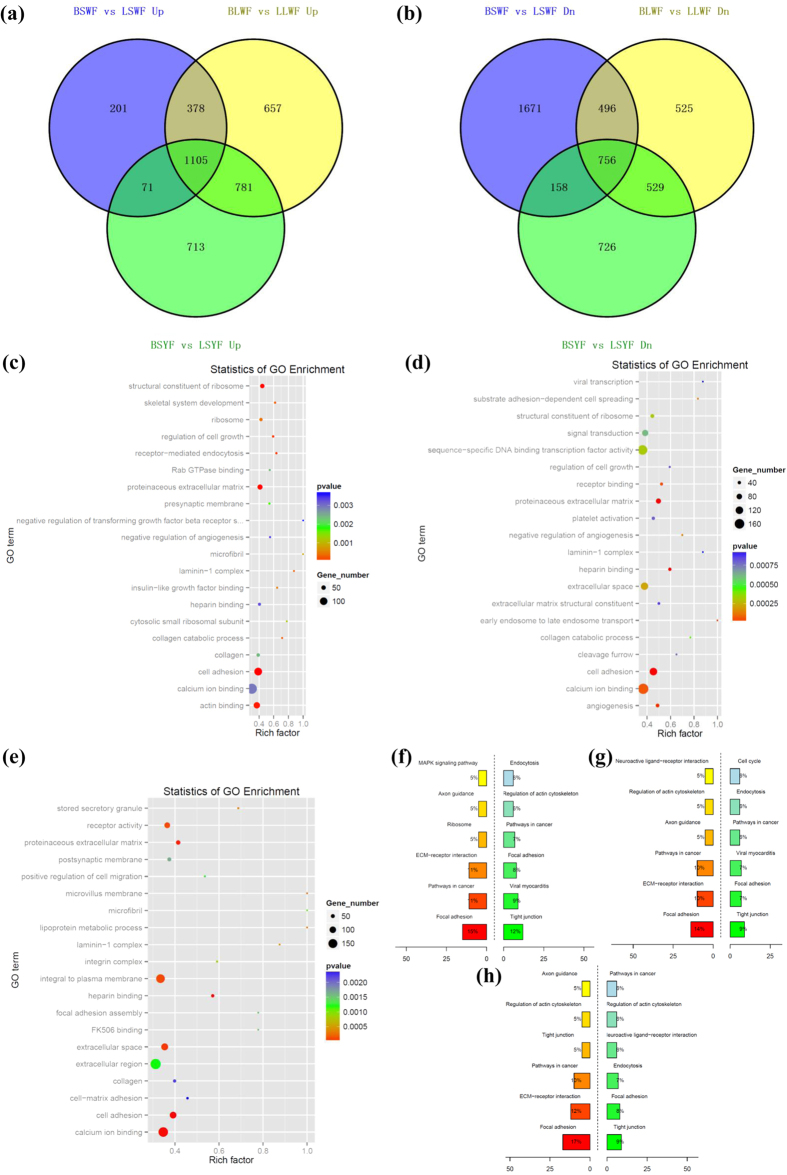
Analysis of differentially expressed genes during the period egg-laying and broody goose follicles. (**a**) Up-regulated genes of SWF, LWF and SYF from egg-laying and broody geese. SWF, small white follicles; LWF, large white follicles; SYF, small yellow follicles; the character L stands for egg-laying goose and the character B stands for broody goose. (**b**) Down-regulated genes of SWF, LWF and SYF from egg-laying and broody geese. (**c**) Analysis of biological functions of GO-enriched genes from LSWF and BSWF. (**d**) Analysis of biological functions of GO-enriched genes from LLWF and BLWF. (**e**) Analysis of biological functions of GO-enriched genes from LSYF and BSYF. (**f**) KEGG pathway enrichment analysis of differentially expressed genes from LSWF and BSWF. (**g**) KEGG pathway enrichment analysis of differentially expressed genes from LLWF and BLWF. (**h**) KEGG pathway enrichment analysis of differentially expressed genes from LSYF and BSYF.

We focused on discovering genes differentially expressed between egg-laying and broody hierarchical follicles, including SWF, LWF, and SYF. Among the 6 libraries, 20,868 genes were detected. Between the LSWF and BSWF libraries, 4,836 significantly differentially expressed genes (*P* < 0.05 and |log_2_ratio | ≥1) composed of 1,755 up-regulated and 3,081 down-regulated genes were identified (Fig. 2a,b). To determine the function of DEGs, we mapped them according to terms of the GO database. A total of 2,918 genes were categorized into the 3 main categories of GO classification, including biological processes, cellular components, and molecular functions (Fig. 2c–e). Among them, the most important biological processes included “transcription, DNA-dependent”, “regulation of transcription, DNA-dependent”, protein transport, apoptosis, and multicellular organismal development. The important cellular components included the membrane, nucleus, and cytoplasm. For molecular function, genes were involved in ATP, protein, zinc ion, and DNA binding. For the LWF libraries, the differentially expressed genes contained 2,921 upregulated genes and 2,306 down-regulated genes in the broody follicles compared to those in the egg-laying follicles. Meanwhile, for the SYF libraries, the differentially expressed genes contained 2,670 upregulated genes and 2,169 down-regulated genes in the broody follicles compared to those in the egg-laying follicles. GO enrichment results of the two libraries show that the cellular components, molecular function, and the biological processes were the same with those of SWF libraries.

For KEGG pathway analysis in LSWF and BSWF libraries, 1,575 genes were mapped onto 265 pathways (Fig. 2f–h). The up-regulated genes were found in the main pathways including focal adhesion (15%), cancer (11%), ECM-receptor interaction (11%), ribosome (5%), axon guidance (5%), and MAPK signaling (5%). The downregulated genes were seen in the tight junction (12%), viral myocarditis (9%), focal adhesion (8%), pathways in cancer (7%), regulation of actin cytoskeleton (6%), and endocytosis (6%). The alteration of gene expression in LWF and SYF libraries was the same with that of SWF libraries.

### Expression analysis of hormone-related genes

**Figure 3:**
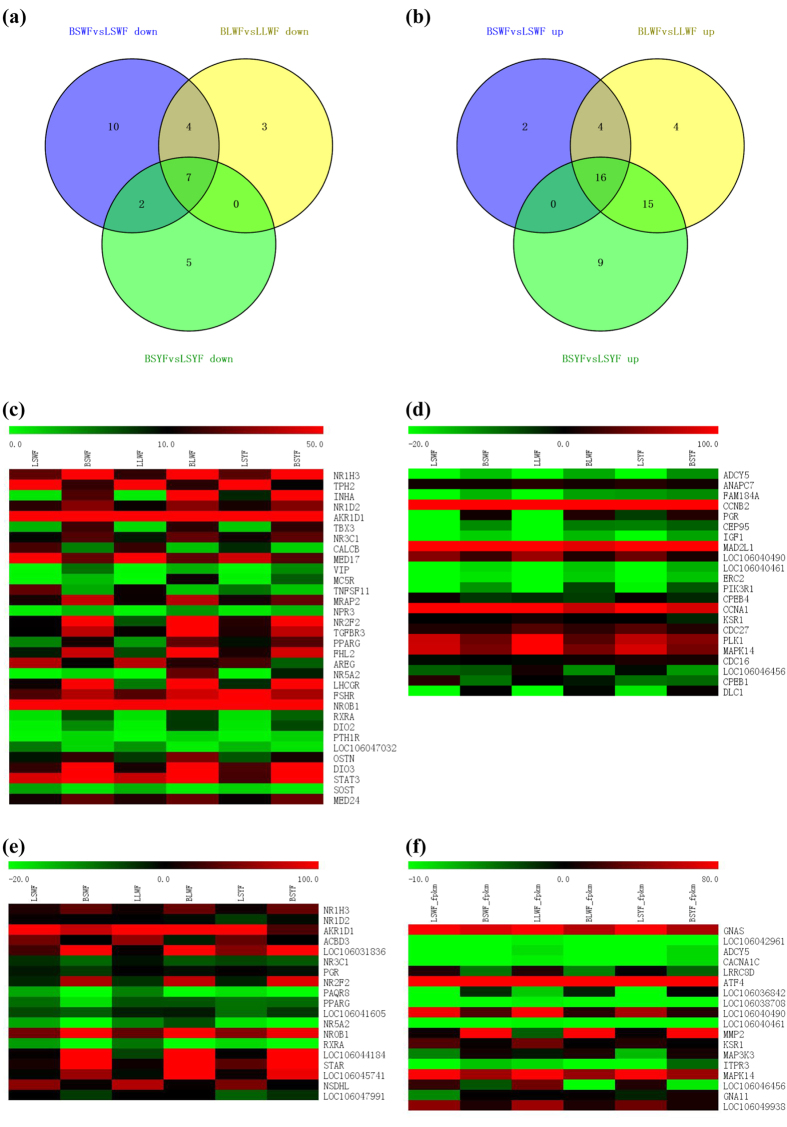
Analysis of differentially expressed hormone-related genes during the period between egg-laying and broody goose follicles. (**a**) Up-regulated hormone-related genes of SWF, LWF and SYF from egg-laying and broody geese. (**b**) Down-regulated hormone-related genes of SWF, LWF and SYF from egg-laying and broody geese. (**c**) Heatmap of hormone-pathway genes of SWF, LWF and SYF from egg-laying and broody geese. (**d**) Heatmap of progesterone-related genes of SWF, LWF and SYF from egg-laying and broody geese. (**e**) Heatmap of steroid-related genes of SWF, LWF and SYF from egg-laying and broody geese. (**f**) Heatmap of GnRH-related genes of SWF, LWF and SYF from egg-laying and broody geese.

Endogenous hormones, including PRL, P2, and E4, are important for the development of follicles and the regulation of broodiness in the goose. In this study, we identified 22, 39, and 40 upregulated hormone-related genes and 23, 14, and 14 downregulated hormone-related genes in SWF, LWF, and SYF of broody geese, compared to these of egg-laying geese (Fig. 3a,b; Table S3). The differentially expressed genes were divided into GnRH-contained, progesterone-contained, and steroid-related groups.

In the GnRH-signaling pathway (Fig. 3c; Table S4), *GNAS, LOC106042961, ADCY5, CACNA1C, LRRC8D, ATF4, LOC106036842, LOC106038708, LOC106040490, LOC106040461, MMP2, KSR1, MAP3K3, ITPR3, MAPK14, LOC106046456, GNA11*, and *LOC106049938* had significantly different expressions in broody geese than those in the egg-laying geese. In the progesterone-signaling pathway (Fig. 3d; Table S5), 22 expression-altered genes were identified in broody follicles, including *ADCY5, ANAPC7, FAM184A, CCNB2, PGR, CEP95, IGF1, MAD2L1, LOC106040490, LOC106040461, ERC2, PIK3R1, CPEB4, CCNA1, KSR1, CDC27, PLK1, MAPK14, CDC16, LOC106046456, CPEB1*, and *DLC1*. In the steroid signaling pathway (Fig. 3e; Table S6), the expression of 19 genes in broody follicles was altered, including *NR1H3, NR1D2, AKR1D1, ACBD3, LOC106031836, NR3C1, PGR, NR2F2, PAQR8, PPARG, LOC106041605, NR5A2, NR0B1, RXRA, LOC106044184, STAR, LOC106045741, NSDHL*, and *LOC106047991*. The above results suggest that the hormones were important for the regulation of broodiness in geese.

### Expression analysis of transcription factors

**Figure 4:**
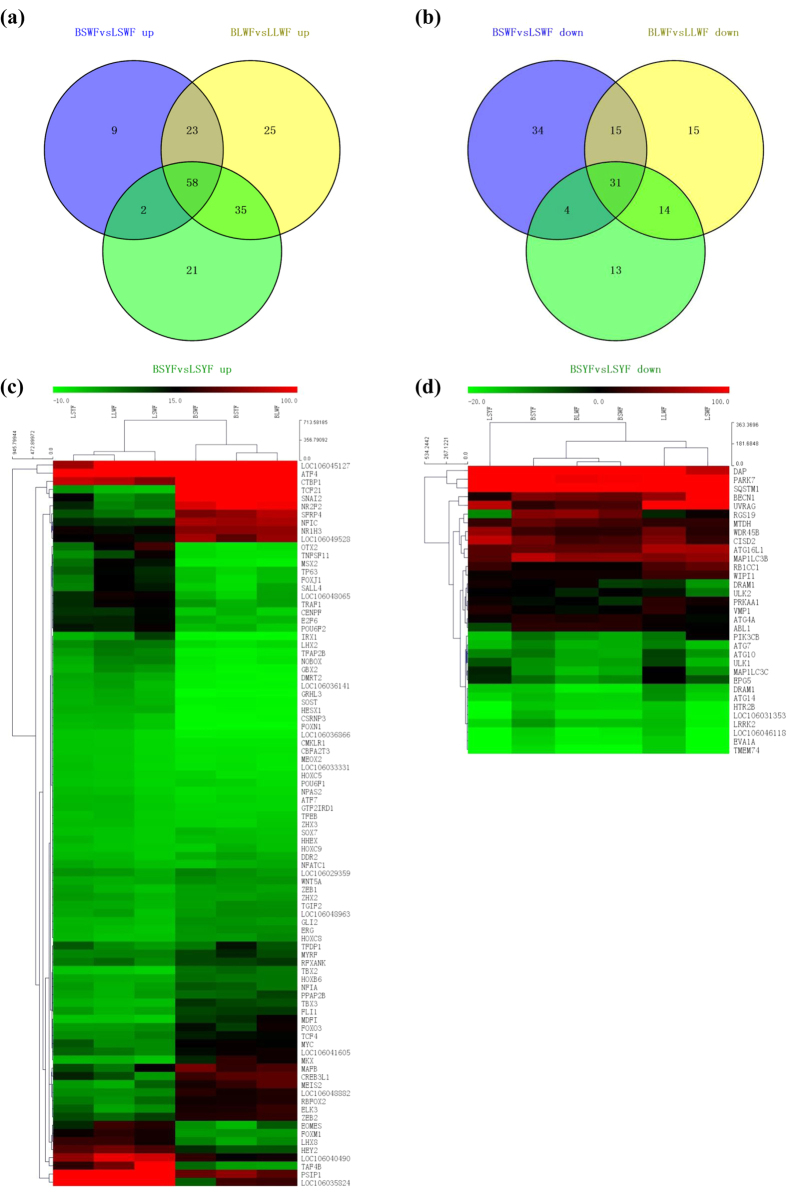
Analysis of differentially expressed transcription factors and autophagy-related genes during the period between egg-laying and broody goose follicles. (**a**) Up-regulated transcription factors of SWF, LWF and SYF from egg-laying and broody geese. (**b**) Down-regulated transcription factors of SWF, LWF and SYF from egg-laying and broody geese. (**c**) Heatmap of transcription factors of SWF, LWF and SYF from egg-laying and broody geese. (**d**) Heatmap of autophagy-related genes of SWF, LWF and SYF from egg-laying and broody geese.

Considering the functional importance of transcription factors, we identified a total of 634 transcription factors expressed in follicles. In the LSWF and BSWF libraries, 176 genes were significantly differentially expressed, including 92 up-regulated and 84 down-regulated (Fig. 4a,b; Table S7). In the LLWF and BLWF libraries, 216 genes were significantly differentially expressed, including 141 up-regulated and 75 down-regulated. In the LSYF and BSYF libraries, 188 genes were significantly differentially expressed, 126 up-regulated, and 62 down-regulated. Together, 58 transcription factors were upregulated and 31 transcription factors downregulated in broody follicles compared with those in egg-laying follicles.

Of the 58 upregulated transcription factors (Fig. 4c, Table S7), 14 were involved in the regulation of autophagy, including *PPAP2B, NR1H3, MYC, ATF4, FOXO3, SFRP4, CTBP1, NT5A, NFATC1, GLI2, NPAS2, CMKLR1, RBFOX2*, and *TFEB* among which *FOXO3* and *LOC106033331* were redox-related genes belonging to forkhead box family. Interestingly, in this group there were 14 homeobox genes, including *HHEX, MEIS2, MEOX2, ZHX2, ZEB2, MKX, ZEB1, ZHX3, TGIF2, HOXB6, POU6F1, HOXC5, LOC106048882, HOXC8*, and *HOXC9*, which were important for the development of the ovum.

Of the 31 downregulated transcription factors (Fig. 4c, Table S7), 10 were homeobox genes including *GBX2, IRX1, HESX1, LHX2, MSX2, POU6F2, LHX8, OTX2, NOBOX*, and *LOC106048065*, which are associated with ovarian follicle development and the regulation of the occurrence of autophagy, and 3 downregulated genes, including *TAF4B, TNFSF11*, and *TP63*, are likely autophagy-regulatory genes. These results show that the autophagy and endogenous redox levels might have a role in regulating the goose broodiness.

### Expression analysis of autophagy-related genes

Based on the GO database, we identified 33 autophagy-related genes, including *BECN1, MAP1LC3B, ATGs, PIK3CB*, and others (Fig. 4d, Table S8). The expression of *PIK3CB, MAP1LC3B*, and *BECN1* was upregulated in the SYF of the broody goose, which was confirmed by qRT-PCR. These results suggest that the autophagy-related genes might play roles in regulating goose broodiness.

### Analysis of the Endogenous hormones level

**Figure 5:**
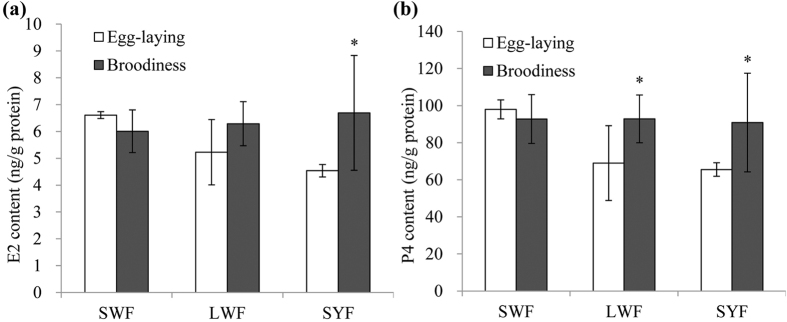
Contents of reproductive endocrine hormones, including E2 (**a**) and P4 (**b**) of SWF, LWF and SYF from egg-laying and broody geese. *Values significantly different (*P* < 0.05) from those in egg-laying geese.

Hormones, including P4 and E2, play regulatory role in goose broodiness. We found that the levels of E2 increased significantly in the SYF of the broody goose, but there was no difference observed in the SWF and LWF during egg-laying and broody stages (Fig. 5a). P4 levels were elevated in the LWF and SYF during the broody stage compared to those of the egg-laying stage (Fig. 5b). The results indicate that P4 and E2 were associated with the broodiness.

### Analysis of autophagy levels

**Figure 6:**
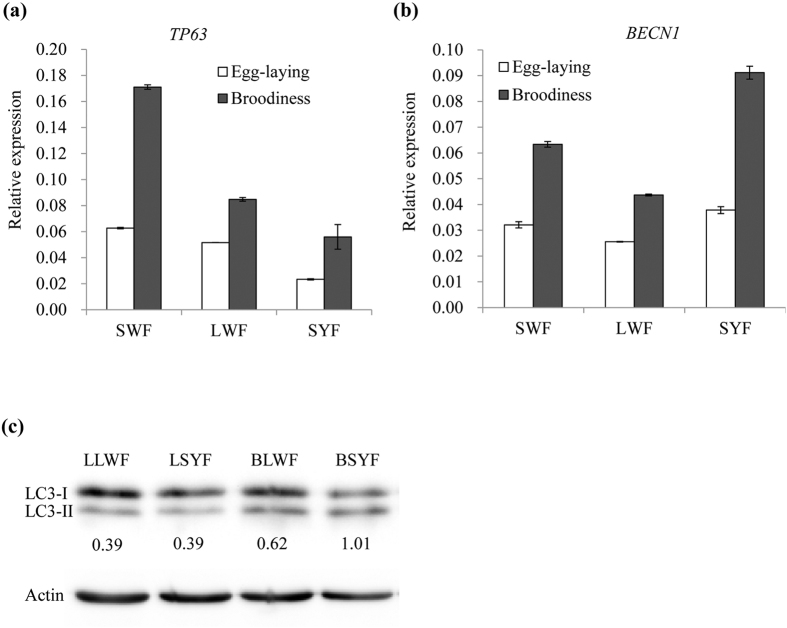
Analysis of autophagy levels in goose follicles. (**a**) Validation of the different expression of TP63 by qRT-PCR. (**b**) Validation of the different expression of BECN1 by qRT-PCR. (**c**) Analysis of autophagic marker LC3 protein in SWF, LWF and SYF from egg-laying and broody geese using Western blot method.

Our previous study showed that autophagy was associated with broodiness in geese^7^. Combined with the differential expression results from this study, we postulated that the autophagy played a critical role in regulating broodiness in geese. We confirmed the expression of autophagy-related genes, including *TP63* and *BECN1*, by qRT-PCR (Fig. 6a,b). We also detected the level of LC3 protein, a biomarker of autophagy, in the follicles of different sizes during both egg-laying and broody stages (Fig. 6c). Since LC3-II levels increased in the broody follicles, it suggests that the autophagy levels were raised at the broody stage in geese.

## Discussion

Broody behavior of birds is significant for their reproduction, but it affects the egg production with the degeneration of follicles. During the broody stage, LYF, SYF, and LWF are significantly reduced in geese and rapidly increase after broodiness^6^. Some studies have shown that the degeneration of follicles is associated with autophagy, apoptosis and homeostasis imbalances, including the altered hormones and ROS level^7^. Furthermore, the transcriptomic results indicate that the development or degeneration of follicles requires the coordinated actions of abundant genes controlled at the transcriptional and posttranscriptional levels^27^. In addition, miRNAs also function in the regulation of follicular development to regulate the bird broodiness^18^. However, previous studies mainly focused on the gene expressions of the ovary or the mixed follicles at the egg-laying and broody stages; they did not extensively analyze the regulatory mechanism of goose broodiness in follicles of different grades.

In the current study, we provided new insight into goose follicular transcriptomes at egg-laying and broody stages by RNA-seq transcriptome analysis. We performed transcriptome profiling of follicles of different grades (SWF, LWF, and SYF) during egg-laying and broody stages in geese to identify genes that are differentially expressed. Many hormone-related genes, autophagy- and redox-related transcription factors were identified.

Also, the hormones, such as progesterone and estradiol function in regulating the development of follicles, ovulation, and sexual behaviors in birds^28^ and the altered levels of reproductive hormones are associated with broodiness^7^. In this study, we demonstrated that progesterone and estradiol increased and the expressions of hormone-related genes were altered in the broody follicles. These results indicate that hormones played an important role in regulating broodiness in geese. The cyclins are involved in regulating the follicular size. CCNB2, as an important factor for cellular divisions, rose sharply to increase follicle sizes^29^. CPEB1 is involved in Cyclin B translation and meiotic resumption to regulate the development of oocytes^30^. In this study, we found that *CCNB2, CCNA1, CPEB1, CDC27*, and *CDC16* decreased significantly in the broody follicles, suggesting that the development of follicles was inhibited during the broody stage.

Insulin and insulin-like growth factor 1 (IGF1) could stimulate granulosa cells producing estradiol^31^. Correspondingly, an elevated level of IGF1 and increased contents of estradiol were detected in the broody follicles in geese. MAPKs play important roles in follicle development^32^. The expression of *MAPK14* was reduced during the broody stage in geese. Polo-like kinase 1 (Plk1), involved in meiotic arrest of oocytes^33^, also decreased in the broody follicles in geese. An increase in KSR1 expression leads to an increase in proliferative capacity of cells^34^. Conversely, the expression of KSR1 was downregulated in the broody follicles, resulting in the bad follicular development. Our results suggest that broodiness affected the development of follicles.

Autophagy is involved in both cell survival and cell death^35^. Our previous study showed that autophagy was associated with broodiness in geese^7^. In the present study, we detected an elevated level of autophagy in the broody follicles and many autophagy-related genes, such as *BECN1, TP63*, and *ATGs*, alerted their expression in the broody follicles. ATF4 regulates a set of autophagy genes, including *Atg16l1, Map1lc3b, Atg12, Atg3, Beclin1*, and *Gabarapl2*, implicated in the formation, elongation, and function of the autophagosome to control autophagy and respond to severe hypoxia^36^. MYC also functions in induction of autophagy^37^.

Increased TNFSF11 production is associated with increased oxidative stress in osteoblasts^38^. Akt/PKB activation blocks FoxO3 activation and autophagy, and this effect is not prevented by rapamycin. FoxO3, blocked by Akt/PKB, controls the expression of autophagy-related genes, including LC3 and Bnip3, to regulate autophagy^39^. The overproduction of chemerin (CMKLR1) in mice reduced the generation of mitochondrial reactive oxygen species (ROS) and increased mitochondrial autophagy, as determined by the increased conversion of LC3-I to LC3-II and higher expression levels of Beclin1 and autophagy-related protein-5 and 7 40,41. TRP63 and TRP73, the members of TRP53 family, can bind and regulate target genes through the same consensus site as TRP53 to activate a global autophagy program^42^. TFEB, having a much broader activity, controls a number of autophagy genes involved in multiple crucial steps of the autophagic pathway such as autophagosome formation, autophagosome lysosome fusion and lysosome-mediated degradation of the autophagosomal content. The regulation of TFEB activity, not affected by rapamycin, is similar to that of FOXO3a, and TFEB and FOXO3a act in parallel mechanisms to regulate autophagy^39^,^44^.

In this study, many homeobox domain genes, involved in the regulation of patterns of anatomical development, were differentially expressed in broody goose follicles. Newborn ovary homeobox (NOBOX), an oocyte-specific transcription factor, is preferentially expressed in the germ cells throughout folliculogenesis^45^,^46^. Female mice lacking NOBOX are infertile due to postnatal oocyte loss and a disrupted transition in follicular development from primordial to primary follicle^46^. In the broody goose, the NOBOX genes were downregulated in LWF and SYF compared to those in egg-laying goose. Combined with the sharply reduced numbers of LWF and SYF in geese during broodiness, we suggest that the altered expression of homeobox genes controlled the follicular development during broodiness.

In the present study, a comparative RNA-Seq transcriptomics approach led to the identification of hundreds of genes differentially expressed in goose follicles during egg-laying and broody stages. Many of them functioned predictably on the pathways of hormone response, follicular development, autophagy, and oxidation. Furthermore, the rise of broodiness in geese was accompanied by the altered levels of progesterone and estradiol and the elevated autophagy levels of follicles marked by LC3-II to LC3-I. Overall, this study provided novel insights into broodiness in birds and demonstrated that RNA-Seq is a powerful tool for examining the molecular mechanism in regulating broodiness.

## Materials and Methods

### Ethics statement

All experimental protocols employed in the present study that relate to animal experimentation were performed in accordance with Measures for the Administration of Affairs Concerning Experimental Animal of Zhejiang Province, China (Approved by the Zhejiang Provincial Government in 2009 and promulgated by Decree No. 263). All animal experiments of this study were approved by the Animal Care and Use Committee of the Zhejiang Agriculture and Forestry University (Lin’an, Zhejiang, China), in order to ensure compliance with international guidelines for animal welfare.

### Animals, feeding, and follicle collection

A total of 6 Zhedong white geese, including 3 broody and 3 egg-laying geese, were collected from the breeding farm of Zhejiang Xiangshan Animal Husbandry Bureau. According to the diameter of follicles, the SWF (2–4 mm), LWF (4–6 mm), and SYF (6–8 mm) of geese were sampled, frozen in liquid nitrogen, and then stored at −80 °C until use.

### Construction of mRNA library and sequencing

Total RNAs of goose follicles were extracted using Trizol reagent (Invitrogen, CA, USA) following the manufacturer’s procedure. Their quantity and purity were analyzed by the Bioanalyzer 2100 and RNA 6000 Nano LabChip Kit (Agilent, CA, USA) with RNA integrity >7.0. Approximately 5-μg total RNA of each sample was subjected to isolating Poly (A) mRNA with poly-T oligo attached magnetic beads (Invitrogen). Following purification, the mRNA is fragmented into small pieces using divalent cations under elevated temperature. Then the cleaved RNA fragments were constructed into the cDNA library according to the protocol for the Illumina RNA ligation based method (Illumina, San Diego, USA). We performed the single end sequencing on an Illumina Hiseq 2500 at the LC Sciences, China, following the vendor’s recommended protocol.

### Sequencing and primary analysis

A cDNA library was sequenced run with Illumina 2500 sequence platform. Using the Illumina paired-end RNA-seq approach, we sequenced the transcriptome of SWF, LWF, and SYF sampled during egg-laying and broody stages, respectively. Prior to assembly, the low quality reads (containing sequencing adaptors, sequencing primers, or nucleotide with q quality scored lower than 20) were removed. The raw sequence data were submitted to the NCBI Short Read Archive with accession numbers.

### RNA-seq reads mapping

We aligned the reads of 18 samples to the UCSC (http://genome.ucsc.edu/) goose reference genome using Tophat package, which initially removed a portion of the reads based on quality information accompanying each read and then mapped the reads to the reference genome of goose. Tophat allows multiple alignments per read (up to 20 by default) and a maximum of two mismatches when mapping the reads to the reference.

### Transcript abundance estimation and differentially expressed testing

The aligned reads were processed by Cufflinks, which use the normalized RNA-seq fragment counts to measure the relative abundances of the transcripts. The unit of measurement is fragment per kilobase of exon per million fragments mapped (FPKM). The reference GTF annotation used in Cuffinks was downloaded from the UCSC database. Cufflink was used to de novo assemble the transcriptome. The second, Cuffmerge was used to co-merge all transcripts of samples to generate unique transcripts. The downloaded UCSC GTF file was passed to Cuffdiff along with the original alignment (SAM) files produced by Tophat. Cuffdiff re-estimates the abundance of the transcripts listed in the GTF file using alignments from the SAM file and concurrently tests for different expression. Only the comparisons with “p value” less than 0.05 and status marked as “OK” in the Cuffdiff output were regarded as showing differential expression.

The differentially expressed genes, including transcription factor, hormone related genes, and autophagy-related genes, were analyzed using MeV software (http://www.tm4.org/mev.html), and confirmed partially by qRT-PCR methods. Gene Ontology (GO) of differential genes was carried out using GeneOntology database (http://www.geneontology.org/). KEGG (Kyoto Encyclopedia of Genes and Genomes) pathway analysis was analyzed by KEGG database.

### Analysis of P4 and E2

P4 and E2 in the different grade follicles (SWF, LWF, and SYF of egg-laying and broody goose) were analyzed by using a goose progesterone assay kit (H089), and goose estradiol assay kit (H102), respectively (Nanjing Jiancheng Bioengineering Institute, Nanjing, China). The samples were processed according to the previous report^7^. All the analyses were carried out according to the kit protocols in three biological repeats. Statistical analysis was carried out by Microsoft Excel. Variance was compared based on t-test and the significance was determined when *P* < 0.05.

### Analysis of autophagy by western blot

Levels of the goose follicular LC3 protein, a marker of autophagy, were detected using rabbit polyclonal to LC3 (Abcam) and goat anti-rabbit IgG and ECL. Western blot was carried out according to the previous report^7^,^47^.

## Additional Information

**How to cite this article**: Yu, J. *et al*. Transcriptome analysis of follicles reveals the importance of autophagy and hormones in regulating broodiness of Zhedong white goose. *Sci. Rep.* **6**, 36877; doi: 10.1038/srep36877 (2016).

**Publisher’s note:** Springer Nature remains neutral with regard to jurisdictional claims in published maps and institutional affiliations.

## Supplementary Material

Supplementary Information

## Figures and Tables

**Table 1:** Characteristics of the reads from 18 different grade follicle libraries in geese.

Sample	Raw data		Valid data		Valid ratio %	Q20%	Q30%	GC content %
	Read	Base	Read	Base	Read			
BSWF1	30417008	3.80G	29846346	3.73G	98.12	98.88	87.85	47.5
BSWF2	34611222	4.33G	33732832	4.22G	97.46	98.97	91.76	47
BSWF4	34834864	4.35G	34397820	4.30G	98.75	98.96	91.3	47
BLWF1	66767248	8.35G	65818230	8.23G	98.58	98.8	90.72	47.5
BLWF3	59948326	7.49G	59002520	7.38G	98.42	99.07	91.93	47.5
BLWF4	47732516	5.97G	47209250	5.90G	98.9	98.63	89.86	47.5
BSYF1	58530418	7.32G	57284462	7.16G	97.87	98.76	90.4	48
BSYF3	100673900	12.58G	99189858	12.40G	98.53	98.99	91.53	47.5
BSYF4	35887808	4.49G	35180814	4.40G	98.03	98.85	90.74	47.5
LSWF11	51813516	6.48G	50380606	6.30G	97.23	99.01	91.65	47.5
LSWF12	47105842	5.89G	46002046	5.75G	97.66	99.19	92.97	46
LSWF13	45794496	5.72G	44107068	5.51G	96.32	99.12	92.16	45
LLWF11	34848262	4.36G	34053388	4.26G	97.72	98.97	82.37	45.5
LLWF12	32945100	4.12G	32305532	4.04G	98.06	98.92	82.76	46
LLWF13	35216142	4.40G	34394692	4.30G	97.67	98.93	84.29	46
LSYF11	34853002	4.36G	33811710	4.23G	97.01	98.87	83.87	45
LSYF12	32172948	4.02G	31380150	3.92G	97.54	99.07	85.21	43.5
LSYF13	30831188	3.85G	30141568	3.77G	97.76	99.01	86.27	43.5

**Table 2:** Distribution of transcript coverage.

Coverage	BSWF1	BSWF2	BSWF4	BLWF1	BLWF3	BLWF4	BSYF1	BSYF3	BSYF4	LSWF11	LSWF12	LSWF13	LLWF11	LLWF12	LLWF13	LSYF11	LSYF12	LSYF13
0–1	58.94%	54.88%	57.14%	53.95%	50.98%	57.83%	53.56%	52.22%	59.14%	52.41%	51.76%	51.84%	55.18%	56.42%	56.42%	56.70%	57.07%	58.18%
2–5	10.04%	11.96%	9.29%	9.08%	10.68%	8.02%	9.55%	8.57%	9.08%	12.53%	6.31%	6.87%	13.29%	11.79%	11.97%	13.23%	12.75%	11.63%
6–10	7.43%	7.55%	7.17%	5.95%	7.44%	6.12%	6.18%	5.61%	6.53%	7.84%	4.70%	5.71%	7.97%	7.27%	7.17%	7.19%	7.54%	7.22%
11–15	4.31%	4.62%	4.34%	3.70%	4.19%	4.07%	4.05%	3.41%	4.04%	4.16%	3.73%	4.87%	4.06%	4.03%	3.98%	3.94%	4.15%	4.14%
16–20	3.11%	2.93%	3.21%	2.63%	3.05%	2.92%	2.89%	2.36%	2.92%	2.75%	3.21%	3.71%	2.76%	2.69%	2.56%	2.53%	2.78%	2.85%
21–25	2.17%	2.25%	2.47%	2.22%	2.23%	2.25%	2.27%	1.99%	2.25%	2.06%	2.70%	2.86%	1.87%	1.97%	2.00%	1.89%	2.07%	1.98%
26–30	1.75%	1.77%	1.84%	1.86%	1.83%	1.80%	1.83%	1.69%	1.66%	1.62%	2.35%	2.52%	1.61%	1.59%	1.57%	1.51%	1.60%	1.61%
>30	12.26%	14.05%	14.53%	20.61%	19.61%	17.01%	19.67%	24.16%	14.38%	16.62%	25.25%	21.61%	13.26%	14.25%	14.33%	13.01%	12.03%	12.40%
