# Supplementary material for: Transcriptome analysis of follicles reveals the importance of autophagy and hormones in regulating broodiness of Zhedong white goose
Source: Sci Rep. 2016 Nov 11;6:36877. doi: 10.1038/srep36877 (PMC5105085; doi:10.1038/srep36877)

Transcriptome analysis of follicles reveals the importance of autophagy and hormones in regulating broodiness of Zhedong white goose

Jing Yu#, Yaping Lou#, Ayong Zhao*

Supplemental data

Supplemental Table Legends

Table S1 Analysis of sample correlation

Table S2 Mapped statistical results of 18 libraries

Table S3 Differentially expressed hormone-related genes during the period between egg-laying and broody goose follicles

Table S4 Differentially expressed progesterone-related genes during the period between egg-laying and broody goose follicles

Table S5 Differentially expressed GnRH-related genes during the period between egg-laying and broody goose follicles

Table S6 Differentially expressed steroid-related genes during the period between egg-laying and broody goose follicles

Table S7 Differentially expressed transcription factors during the period between egg-laying and broody goose follicles

Table S8 Analysis of autophagy-related genes during the period between egg-laying and broody goose follicles

Supplemental Figure Legends

Figure S1 Map of gene expression density

Table S1 Analysis of sample correlation

	BSWF1	BSWF2	BSWF4	BLWF1	BLWF3	BLWF4	BSYF1	BSYF3	BSYF4	LSWF11	LLWF11	LLWF12	LLWF13	LSYF11	LSYF12	LSYF13
BSWF1	1.00	0.81	0.89	0.95	0.78	0.86	0.99	0.93	0.93	0.89	0.88	0.83	0.52	0.74	0.90	0.92
BSWF2	0.81	1.00	0.83	0.78	0.83	0.87	0.80	0.84	0.80	0.86	0.70	0.81	0.65	0.76	0.73	0.86
BSWF4	0.89	0.83	1.00	0.84	0.80	0.89	0.88	0.92	0.90	0.87	0.83	0.81	0.53	0.73	0.86	0.89
BLWF1	0.95	0.78	0.84	1.00	0.84	0.88	0.97	0.85	0.83	0.83	0.74	0.80	0.56	0.74	0.77	0.87
BLWF3	0.78	0.83	0.80	0.84	1.00	0.93	0.79	0.81	0.71	0.83	0.56	0.77	0.70	0.80	0.59	0.84
BLWF4	0.86	0.87	0.89	0.88	0.93	1.00	0.86	0.91	0.85	0.88	0.71	0.81	0.61	0.77	0.74	0.90
BSYF1	0.99	0.80	0.88	0.97	0.79	0.86	1.00	0.90	0.90	0.88	0.85	0.83	0.55	0.76	0.87	0.90
BSYF3	0.93	0.84	0.92	0.85	0.81	0.91	0.90	1.00	0.98	0.91	0.92	0.84	0.48	0.71	0.93	0.92
BSYF4	0.93	0.80	0.90	0.83	0.71	0.85	0.90	0.98	1.00	0.89	0.97	0.81	0.43	0.67	0.97	0.88
LSWF11	0.89	0.86	0.87	0.83	0.83	0.88	0.88	0.91	0.89	1.00	0.84	0.90	0.74	0.89	0.84	0.91
LLWF11	0.88	0.70	0.83	0.74	0.56	0.71	0.85	0.92	0.97	0.84	1.00	0.78	0.37	0.62	0.99	0.82
LLWF12	0.83	0.81	0.81	0.80	0.77	0.81	0.83	0.84	0.81	0.90	0.78	1.00	0.77	0.87	0.80	0.89
LLWF13	0.52	0.65	0.53	0.56	0.70	0.61	0.55	0.48	0.43	0.74	0.37	0.77	1.00	0.90	0.37	0.65
LSYF11	0.74	0.76	0.73	0.74	0.80	0.77	0.76	0.71	0.67	0.89	0.62	0.87	0.90	1.00	0.63	0.83
LSYF12	0.90	0.73	0.86	0.77	0.59	0.74	0.87	0.93	0.97	0.84	0.99	0.80	0.37	0.63	1.00	0.85
LSYF13	0.92	0.86	0.89	0.87	0.84	0.90	0.90	0.92	0.88	0.91	0.82	0.89	0.65	0.83	0.85	1.00

Table S2 Mapped statistical results of 18 libraries

Sample	BSWF1	BSWF2	BSWF4	BLWF1	BLWF3	BLWF4	BSYF1	BSYF3	BSYF4	LSWF11	LSWF12	LSWF13	LLWF11	LLWF12	LLWF13	LSYF11	LSYF12	LSYF13
Valid reads	29846346	33732832	34397820	65818230	59002520	47209250	57284462	99189858	35180814	50380606	46002046	44107068	34053388	32305532	34394692	33811710	31380150	30141568
Mapped reads	17479292(58.56%)	21129671(62.64%)	22481263(65.36%)	42519819(64.60%)	38541790(65.32%)	30912946(65.48%)	35860538(62.60%)	63004627(63.52%)	22084002(62.77%)	30716145(60.97%)	32515456(70.68%)	31551474(71.53%)	20867516(61.28%)	22142860(68.54%)	22602311(65.71%)	21832266(64.57%)	20368266(64.91%)	19969215(66.25%)
Unique Mapped reads	17143785(57.44%)	20724990(61.44%)	21961185(63.84%)	41719762(63.39%)	38222735(64.78%)	29960769(63.46%)	35222681(61.49%)	62111769(62.62%)	21789011(61.93%)	30497002(60.53%)	32313399(70.24%)	31155519(70.64%)	20757585(60.96%)	21998760(68.10%)	22451999(65.28%)	21685935(64.14%)	20206389(64.39%)	19749074(65.52%)
Multi Mapped reads	335507(1.12%)	404681(1.20%)	520078(1.51%)	800057(1.22%)	319055(0.54%)	952177(2.02%)	637857(1.11%)	892858(0.90%)	294991(0.84%)	219143(0.43%)	202057(0.44%)	395955(0.90%)	109931(0.32%)	144100(0.45%)	150312(0.44%)	146331(0.43%)	161877(0.52%)	220141(0.73%)
PE Mapped reads	6675685(22.37%)	8212060(24.34%)	8788438(25.55%)	16258350(24.70%)	14963478(25.36%)	11891401(25.19%)	13759154(24.02%)	24229114(24.43%)	8530782(24.25%)	11546176(22.92%)	13731920(29.85%)	13373020(30.32%)	7641051(22.44%)	8820230(27.30%)	8863450(25.77%)	8114038(24.00%)	7562684(24.10%)	7698531(25.54%)
Mapped left reads	9128795(30.59%)	11319942(33.56%)	12059991(35.06%)	22994832(34.94%)	20562860(34.85%)	16837478(35.67%)	19422937(33.91%)	33725938(34.00%)	11908208(33.85%)	16472705(32.70%)	17127156(37.23%)	16700396(37.86%)	9854339(28.94%)	10900843(33.74%)	11164507(32.46%)	10819030(32.00%)	10039426(31.99%)	9895146(32.83%)
Mapped right reads	8350497(27.98%)	9809729(29.08%)	10421272(30.30%)	19524987(29.67%)	17978930(30.47%)	14075468(29.82%)	16437601(28.69%)	29278689(29.52%)	10175794(28.92%)	14243440(28.27%)	15388300(33.45%)	14851078(33.67%)	11013177(32.34%)	11242017(34.80%)	11437804(33.25%)	11013236(32.57%)	10328840(32.92%)	10074069(33.42%)
Reads map to sense	8571547(28.	10375320(30	10990408(31	20905356(31	19122893(32	15044031(31	17626045(30	31124656(31	10908723(31	15257766(30	16122209(35	15613966(35	10348754(30	10968346(33	11189657(32	10811449(31	10085410(32	9863493(32.

strand	72%)	.76%)	.95%)	.76%)	.41%)	.87%)	.77%)	.38%)	.01%)	.28%)	.05%)	.40%)	.39%)	.95%)	.53%)	.98%)	.14%)	72%)
Reads map to antisense strand	8572238(28.72%)	10349670(30.68%)	10970777(31.89%)	20814406(31.62%)	19099842(32.37%)	14916738(31.60%)	17596636(30.72%)	30987113(31.24%)	10880288(30.93%)	15239236(30.25%)	16191190(35.20%)	15541553(35.24%)	10408831(30.57%)	11030414(34.14%)	11262342(32.74%)	10874486(32.16%)	10120979(32.25%)	9885581(32.80%)
Non-splice reads	10471949(35.09%)	12418971(36.82%)	13266012(38.57%)	24668007(37.48%)	22453286(38.05%)	17126860(36.28%)	20636190(36.02%)	38225152(38.54%)	12399487(35.25%)	18667618(37.05%)	17889605(38.89%)	17679604(40.08%)	12202717(35.83%)	12568145(38.90%)	12583623(36.59%)	13047033(38.59%)	13096449(41.73%)	13338847(44.25%)
Splice reads	6671836(22.35%)	8306019(24.62%)	8695173(25.28%)	17051755(25.91%)	15769449(26.73%)	12833909(27.19%)	14586491(25.46%)	23886617(24.08%)	9389524(26.69%)	11829384(23.48%)	14423794(31.35%)	13475915(30.55%)	8554868(25.12%)	9430615(29.19%)	9868376(28.69%)	8638902(25.55%)	7109940(22.66%)	6410227(21.27%)

Table S3 Differentially expressed hormone-related genes during the period between egg-laying and broody goose follicles

gene_id			LSWF	BSWF	LLWF	BLWF	LSYF	BSYF
XLOC_001587	NR1H3	"nuclear receptor subfamily 1, group H, member 3"	24.9	68.9	17.6	72.8	23.8	70.2
XLOC_002168	TPH2	tryptophan hydroxylase 2	55.3	18.3	66.6	16.7	50.9	10.8
XLOC_002616	INHA	"inhibin, alpha"	1.0	22.2	0.7	66.6	8.6	96.4
XLOC_003012	NR1D2	"nuclear receptor subfamily 1, group D, member 2"	17.0	30.9	11.6	31.3	13.7	30.5
XLOC_004062	AKR1D1	"aldo-keto reductase family 1, member D1"	563.8	109.3	449.9	134.0	235.5	61.1
XLOC_004497	TBX3	T-box 3	2.9	19.7	1.4	16.7	2.1	17.7
XLOC_005914	NR3C1	"nuclear receptor subfamily 3, group C, member 1 (glucocorticoid receptor)"	9.8	22.9	9.2	25.4	12.9	24.5
XLOC_007274	CALCB	calcitonin-related polypeptide beta	21.2	5.0	18.6	2.4	8.4	1.8
XLOC_007538	MED17	mediator complex subunit 17	95.6	25.7	55.8	25.0	42.7	23.3
XLOC_007585	VIP	vasoactive intestinal peptide	0.3	5.5	0.2	3.0	0.3	4.6
XLOC_008367	MC5R	melanocortin 5 receptor	0.4	2.7	0.1	12.1	0.5	6.4
XLOC_008534	TNFSF11	"tumor necrosis factor (ligand) superfamily, member 11"	25.4	3.3	12.1	2.3	5.5	2.2
XLOC_008821	MRAP2	melanocortin 2 receptor accessory protein 2	13.9	41.1	12.0	38.9	13.5	25.8
XLOC_009946	NPR3	natriuretic peptide receptor 3	0.7	2.7	0.4	4.0	0.9	2.7
XLOC_010065	NR2F2	"nuclear receptor subfamily 2, group F, member 2"	10.5	97.9	6.7	106.5	15.9	119.6
XLOC_013548	TGFBR3	"transforming growth factor, beta receptor III"	10.0	39.6	11.3	50.4	15.0	39.8
XLOC_014279	PPARG	peroxisome proliferator-activated receptor gamma	4.9	13.3	4.0	25.5	9.4	23.1
XLOC_016060	FHL2	four and a half LIM domains 2	8.9	42.0	7.1	50.3	14.3	43.9
XLOC_016411	AREG	amphiregulin	38.1	11.0	38.9	16.3	21.2	6.3
XLOC_016464	NR5A2	"nuclear receptor subfamily 5, group A, member 2"	0.2	1.5	0.1	25.9	0.3	8.8
XLOC_017817	LHCGR	luteinizing hormonechoriogonadotropin receptor	12.0	49.4	5.2	75.3	8.3	65.9
XLOC_017819	FSHR	follicle stimulating hormone receptor	22.3	37.8	23.1	42.7	53.8	36.4

**XLOC_017990**	NR0B1	"nuclear receptor subfamily 0, group B, member 1"	59.2	142.2	49.3	139.9	71.1	125.1
**XLOC_019017**	RXRA	"retinoid X receptor, alpha"	1.2	7.0	1.1	7.7	1.2	6.1
**XLOC_019544**	DIO2	"deiodinase, iodothyronine, type II"	0.5	4.5	0.5	7.9	0.9	7.1
**XLOC_022118**	PTH1R	parathyroid hormone 1 receptor	0.3	1.5	0.2	1.8	0.5	1.7
**XLOC_022413**	LOC106047032	glucagon family neuropeptides-like	5.4	1.6	4.1	0.6	2.8	1.1
**XLOC_023019**	OSTN	osteocrin	9.3	18.2	7.8	29.7	6.6	13.8
**XLOC_023252**	DIO3	"deiodinase, iodothyronine, type III"	17.9	66.1	13.8	81.0	22.0	84.7
**XLOC_023487**	STAT3	signal transducer and activator of transcription 3 (acute-phase response factor)	44.3	78.4	41.0	109.1	20.8	84.0
**XLOC_024345**	SOST	sclerostin	3.6	1.0	3.0	0.6	1.8	0.7
**XLOC_025448**	MED24	mediator complex subunit 24	13.3	24.8	15.5	25.9	11.1	26.7

Table S4 Differentially expressed progesterone-related genes during the period between egg-laying and broody goose follicles

gene_id			LSWF	BSWF	LLWF	BLWF	LSYF	BSYF
**XLOC_000297**	MMS19	"MMS19 homolog, cytosolic iron-sulfur assembly component"	11.4	16.4	13.9	15.3	9.2	15.8
**XLOC_000357**	MAPK8	mitogen-activated protein kinase 8	15.0	8.6	14.8	8.6	14.9	8.2
**XLOC_000483**	CDK1	cyclin-dependent kinase 1	112.4	94.7	193.9	85.0	302.4	161.2
**XLOC_000669**	CPEB2	cytoplasmic polyadenylation element binding protein 2	1.9	2.2	1.3	3.0	2.6	2.4
**XLOC_000704**	ANAPC4	anaphase promoting complex subunit 4	12.8	13.4	16.2	13.0	22.1	14.6
**XLOC_001073**	CFAP44	cilia and flagella associated protein 44	0.5	0.2	0.3	0.2	0.1	0.1
**XLOC_002383**	HSP90AB1	"heat shock protein 90kDa alpha (cytosolic), class B member 1"	31.5	26.9	27.4	30.0	18.3	31.5
**XLOC_002488**	AKT3	v-akt murine thymoma viral oncogene homolog 3	11.1	7.8	9.1	9.1	9.5	7.8
**XLOC_002599**	ADCY5	adenylate cyclase 5	0.0	0.2	0.0	0.1	0.4	0.4
**XLOC_002779**	ADCY5	adenylate cyclase 5	1.6	4.4	1.4	4.5	2.5	5.1
**XLOC_003902**	BICD1	bicaudal D homolog 1 (Drosophila)	6.2	2.9	6.7	2.7	2.8	1.6
**XLOC_003915**	BRAF	"B-Raf proto-oncogene, serinethreonine kinase"	16.7	4.5	10.2	5.2	4.9	3.4
**XLOC_004136**	ANAPC5	anaphase promoting complex subunit 5	73.1	48.1	54.7	54.9	47.1	59.1
**XLOC_004348**	ANAPC7	anaphase promoting complex subunit 7	45.4	26.7	51.5	26.6	40.1	23.7
**XLOC_004416**	MAPK1	mitogen-activated protein kinase 1	100.3	87.8	126.0	90.7	130.8	92.5
**XLOC_004703**	RPS6KC1	"ribosomal protein S6 kinase, 52kDa, polypeptide 1"	22.7	17.6	24.6	17.6	19.8	18.1
**XLOC_005136**	PIK3CA	"phosphatidylinositol-4,5-bisphosphate 3-kinase, catalytic subunit alpha"	9.9	9.3	12.8	11.4	11.8	8.8
**XLOC_005503**	ANAPC10	anaphase promoting complex subunit 10	26.5	10.8	24.1	10.1	25.5	12.1
**XLOC_005660**	FAM184A	"family with sequence similarity 184, member A"	17.5	5.5	15.6	5.2	13.5	5.3
**XLOC_005898**	CDC23	cell division cycle 23	11.4	8.7	11.8	9.0	9.6	9.3
**XLOC_006129**	MAPK9	mitogen-activated protein kinase 9	6.2	8.0	5.7	9.1	9.0	9.5

**XLOC_006279**	LOC106032954	phosphatidylinositol 3-kinase regulatory subunit alpha-like	0.0	0.1	0.0	0.1		
**XLOC_006280**	PIK3R3	"phosphoinositide-3-kinase, regulatory subunit 3 (gamma)"	33.3	18.4	21.2	28.7	11.1	21.8
**XLOC_006661**	ADCY9	adenylate cyclase 9	11.8	15.5	11.9	16.6	12.5	15.5
**XLOC_006696**	NDE1	nudE neurodevelopment protein 1	52.6	39.7	78.6	40.2	66.9	46.0
**XLOC_006899**	MAD1L1	MAD1 mitotic arrest deficient-like 1 (yeast)	85.6	14.0	42.6	15.1	15.7	14.6
**XLOC_006952**	CCNB2	cyclin B2	776.6	340.3	1362.7	245.4	1156.6	304.9
**XLOC_007310**	PRKACB	"protein kinase, cAMP-dependent, catalytic, beta"	57.0	53.3	41.6	57.6	54.4	62.4
**XLOC_007458**	PGR	progesterone receptor	11.6	26.5	10.4	30.9	20.9	30.0
**XLOC_008120**	CEP95	centrosomal protein 95kDa	17.7	8.6	16.4	8.1	15.5	8.8
**XLOC_009069**	GNAI1	"guanine nucleotide binding protein (G protein), alpha inhibiting activity polypeptide 1"	26.6	49.9	22.5	59.7	23.4	58.9
**XLOC_009350**	PRKX	"protein kinase, X-linked"	1.9	2.0	1.0	2.5	1.1	2.3
**XLOC_009448**	TNRC6B	trinucleotide repeat containing 6B	8.4	5.0	7.9	4.7	6.6	4.2
**XLOC_009460**	IGF1	insulin-like growth factor 1 (somatomedin C)	0.2	3.4	0.1	3.3	0.7	3.4
**XLOC_010127**	IGF1R	insulin-like growth factor 1 receptor	9.1	7.9	8.2	9.6	14.1	7.8
**XLOC_010176**	ADCY2	adenylate cyclase 2 (brain)	1.5	3.0	1.5	3.0	2.7	4.3
**XLOC_010698**	CEP83	centrosomal protein 83kDa	55.1	22.2	41.9	25.2	28.1	29.6
**XLOC_010924**	ANAPC2	anaphase promoting complex subunit 2	47.2	32.5	37.6	40.0	25.0	33.7
**XLOC_011020**	AKT1	v-akt murine thymoma viral oncogene homolog 1	21.5	32.7	20.1	43.0	16.7	36.6
**XLOC_011737**	RPS6KA3	"ribosomal protein S6 kinase, 90kDa, polypeptide 3"	6.8	12.9	7.6	19.8	18.5	15.1
**XLOC_011856**	SLMAP	sarcolemma associated protein	35.8	26.6	32.7	27.4	41.9	27.4
**XLOC_012026**	ADCY3	adenylate cyclase 3	0.1	1.9	0.1	1.3	0.1	1.5
**XLOC_012226**	KRAS	Kirsten rat sarcoma viral oncogene homolog	12.7	10.0	8.0	11.0	14.9	13.0
**XLOC_012627**	MAD2L1	MAD2 mitotic arrest deficient-like 1 (yeast)	237.4	127.2	352.4	107.3	396.7	183.8
**XLOC_012916**	RPS6KL1	ribosomal protein S6 kinase-like 1	3.2	6.3	3.9	6.9	2.5	5.7
**XLOC_013015**	PLEKHA1	"pleckstrin homology domain containing, family A (phosphoinositide binding specific)	17.1	26.1	26.5	29.5	28.3	25.5

		member 1"						
**XLOC_013151**	MOS	v-mos Moloney murine sarcoma viral oncogene homolog	1811.<br>8	2874.<br>0	1753.2	4117.<br>5	1802.1	3505.<br>0
**XLOC_013352**	ANAPC13	anaphase promoting complex subunit 13	53.2	33.6	42.3	33.9	41.8	47.1
**XLOC_013492**	CEP70	centrosomal protein 70kDa	85.5	36.3	86.3	44.0	63.1	29.3
**XLOC_013619**	RPS6KA2	"ribosomal protein S6 kinase, 90kDa, polypeptide 2"	0.6	0.4	0.2	0.4	0.3	0.5
**XLOC_013749**	ADCY7	adenylate cyclase 7	9.9	10.0	7.5	9.9	6.4	10.5
**XLOC_014312**	RAF1	"Raf-1 proto-oncogene, serinethreonine kinase"	47.6	54.8	69.9	50.3	75.6	51.9
**XLOC_014387**	MAD2L2	MAD2 mitotic arrest deficient-like 2 (yeast)	156.5	93.9	195.9	93.5	166.9	118.5
**XLOC_014516**	PIK3CB	"phosphatidylinositol-4,5-bisphosphate 3-kinase, catalytic subunit beta"	22.5	6.8	7.0	8.7	5.6	8.9
**XLOC_014741**	LOC106040<br>490	mitogen-activated protein kinase 11-like	90.8	41.1	97.3	30.4	67.8	25.7
**XLOC_014742**	LOC106040<br>461	mitogen-activated protein kinase 12-like	0.8	2.5	0.5	2.2	0.9	2.4
**XLOC_014835**	ERC2	ELKSRAB6-interactingCAST family member 2	7.1	1.6	5.4	1.7	2.8	1.0
**XLOC_015731**	SYCP1	synaptonemal complex protein 1	2.0	0.5	1.3	0.9	0.9	0.4
**XLOC_016355**	MAPK10	mitogen-activated protein kinase 10	0.1	0.2	0.4	0.7	0.2	0.3
**XLOC_016479**	PIK3CG	"phosphatidylinositol-4,5-bisphosphate 3-kinase, catalytic subunit gamma"	2.1	3.3	1.4	3.1	3.3	3.1
**XLOC_016575**	PIK3R1	"phosphoinositide-3-kinase, regulatory subunit 1 (alpha)"	2.6	8.1	1.6	12.6	6.4	9.6
**XLOC_016578**	CCNB1	cyclin B1	9238.<br>8	4021.<br>8	10301.<br>2	3518.<br>2	10332.<br>4	3710.<br>6
**XLOC_016786**	ANAPC1	anaphase promoting complex subunit 1	160.6	52.7	94.1	44.1	66.0	50.1
**XLOC_017026**	CCNA2	cyclin A2	45.8	27.0	59.5	25.0	61.8	43.4
**XLOC_017196**	INS	insulin			0.2	0.0		
**XLOC_017198**	IGF2	insulin-like growth factor 2	55.1	66.1	48.9	64.9	32.4	89.0
**XLOC_017387**	CPEB4	cytoplasmic polyadenylation element binding protein 4	43.7	15.6	33.6	13.1	28.1	13.3

**XLOC_017450**	LOC106042 860	guanine nucleotide-binding protein G(i) subunit alpha-2	201.3	242.6	136.3	299.1	88.6	276.3
**XLOC_017666**	RPS6KA6	"ribosomal protein S6 kinase, 90kDa, polypeptide 6"	6.6	4.8	7.1	5.0	10.0	4.4
**XLOC_017810**	SPDYA	speedyRINGO cell cycle regulator family member A	10.4	3.4	8.0	3.3	6.5	3.0
**XLOC_017902**	CCNA1	cyclin A1	515.0	142.6	480.0	95.7	327.5	101.3
**XLOC_018213**	IFT80	intraflagellar transport 80	81.3	46.5	56.2	70.5	53.8	68.9
**XLOC_018405**	MAP2K1	mitogen-activated protein kinase kinase 1	35.4	37.6	64.8	37.1	104.0	44.5
**XLOC_018447**	MAP2K1	mitogen-activated protein kinase kinase 1	191.0	132.2	295.0	158.2	205.6	156.9
**XLOC_018627**	KSR1	kinase suppressor of ras 1	39.3	19.5	44.8	18.4	26.6	13.5
**XLOC_019244**	PIK3CD	"phosphatidylinositol-4,5-bisphosphate 3-kinase, catalytic subunit delta"	1.8	2.8	1.1	3.3	1.3	2.4
**XLOC_020387**	TOX3	TOX high mobility group box family member 3	2.4	0.9	1.7	0.4	0.6	0.5
**XLOC_020457**	TBCK	TBC1 domain containing kinase	20.8	13.5	32.7	10.3	47.1	12.2
**XLOC_020693**	KSR2	kinase suppressor of ras 2	0.0	0.0	0.1	0.1	0.1	0.1
**XLOC_020821**	FZR1	fizzycell division cycle 20 related 1	31.8	20.7	32.2	21.6	18.8	18.6
**XLOC_021144**	CDC27	cell division cycle 27	50.5	31.1	69.5	35.3	59.0	29.9
**XLOC_021196**	PLK1	polo-like kinase 1	118.4	62.1	151.6	51.2	99.6	68.3
**XLOC_021324**	MAPK14	mitogen-activated protein kinase 14	119.7	67.5	138.1	64.8	109.7	62.4
**XLOC_021325**	MAPK13	mitogen-activated protein kinase 13	0.8	0.8	1.5	0.7	1.0	0.5
**XLOC_021578**	ADCY8	adenylate cyclase 8 (brain)	0.6	1.1	0.3	1.0	0.3	1.0
**XLOC_021684**	CDC16	cell division cycle 16	37.7	20.2	36.4	23.8	37.3	25.6
**XLOC_021702**	ADCY1	adenylate cyclase 1 (brain)	0.1	0.0	0.0	0.0		
**XLOC_021703**	LOC106046 456	uncharacterized LOC106046456	30.9	13.0	45.7	6.7	24.9	4.3
**XLOC_021977**	PIK3R5	"phosphoinositide-3-kinase, regulatory subunit 5"	2.3	2.9	2.7	4.1	1.3	3.4
**XLOC_022311**	HSP90AA1	"heat shock protein 90kDa alpha (cytosolic), class A member 1"	1017.5	759.3	972.2	780.5	935.0	1066.1

**XLOC_022817**	CPEB1	cytoplasmic polyadenylation element binding protein 1	53.8	10.7	37.4	16.1	16.9	8.1
**XLOC_023338**	RPS6KA1	"ribosomal protein S6 kinase, 90kDa, polypeptide 1"	52.3	31.9	49.7	32.4	33.3	27.4
**XLOC_024071**	STOML2	stomatin (EPB72)-like 2	87.1	78.6	109.0	80.2	74.0	80.9
**XLOC_024137**	DLC1	DLC1 Rho GTPase activating protein	9.3	19.9	8.6	17.4	7.4	19.1
**XLOC_025259**	CCNB3	cyclin B3	33.7	31.0	38.1	30.9	23.1	43.0
**XLOC_025377**	LOC106049631	RAC-beta serinethreonine-protein kinase-like	62.7	100.7	79.6	101.0	57.7	100.0
**XLOC_025534**	GNAI3	"guanine nucleotide binding protein (G protein), alpha inhibiting activity polypeptide 3"	276.3	173.6	235.3	193.0	204.8	221.1
**XLOC_025595**	SPDYC	speedyRINGO cell cycle regulator family member C	10.2	2.8	11.7	2.8	3.5	1.8

Table S5 Differentially expressed GnRH-related genes during the period between egg-laying and broody goose follicles

		LSWF	BSWF	LLWF	BLWF	LSYF	BSYF
**GNAS**	GNAS complex locus	156.7	86.2	197.6	74.1	133.4	67.6
**LOC106042961**	cytosolic phospholipase A2 epsilon-like	0.8	0.1	0.7	0.1	0.7	0.1
**ADCY5**	adenylate cyclase 5	1.6	4.4	1.4	4.5	2.5	5.1
**CACNA1C**	"calcium channel, voltage-dependent, L type, alpha 1C subunit"	1.0	5.1	0.7	4.5	2.0	5.0
**LRRC8D**	"leucine rich repeat containing 8 family, member D"	24.6	11.9	20.8	12.1	15.7	9.7
**ATF4**	activating transcription factor 4	144.2	317.7	159.2	335.8	152.3	354.7
**LOC106036842**	mitogen-activated protein kinase kinase kinase 3-like	3.3	14.7	2.2	15.9	1.8	13.6
**LOC106038708**	"calmodulin, striated muscle"	0.7	2.5	0.6	2.4	0.3	2.1
**LOC106040490**	mitogen-activated protein kinase 11-like	90.8	41.1	97.3	30.4	67.8	25.7
**LOC106040461**	mitogen-activated protein kinase 12-like	0.8	2.5	0.5	2.2	0.9	2.4
**MMP2**	matrix metallopeptidase 2	19.2	252.1	6.4	287.8	17.5	268.0
**KSR1**	kinase suppressor of ras 1	39.3	19.5	44.8	18.4	26.6	13.5
**MAP3K3**	mitogen-activated protein kinase kinase kinase 3	9.0	22.8	9.3	26.7	7.0	21.4
**ITPR3**	"inositol 1,4,5-trisphosphate receptor, type 3"	2.2	8.7	1.6	7.2	1.7	9.5
**MAPK14**	mitogen-activated protein kinase 14	119.7	67.5	138.1	64.8	109.7	62.4
**LOC106046456**	uncharacterized LOC106046456	30.9	13.0	45.7	6.7	24.9	4.3
**GNA11**	"guanine nucleotide binding protein (G protein), alpha 11 (Gq class)"	8.8	18.1	11.0	19.7	13.1	21.8
**LOC106049938**	son of sevenless homolog 2-like	58.8	25.5	59.7	26.5	48.7	21.7

Table S6 Differentially expressed steroid-related genes during the period between egg-laying and broody goose follicles

gene_id			LSWF	BSWF	LLWF	BLWF	LSYF	BSYF
**XLOC_001587**	NR1H3	"nuclear receptor subfamily 1, group H, member 3"	24.9	68.9	17.6	72.8	23.8	70.2
**XLOC_003012**	NR1D2	"nuclear receptor subfamily 1, group D, member 2"	17.0	30.9	11.6	31.3	13.7	30.5
**XLOC_004062**	AKR1D1	"aldo-keto reductase family 1, member D1"	563.8	109.3	449.9	134.0	235.5	61.1
**XLOC_004710**	ACBD3	acyl-CoA binding domain containing 3	58.1	30.9	68.2	29.8	57.9	31.8
**XLOC_005049**	LOC106031836	"steroid 17-alpha-hydroxylase17,20 lyase"	39.1	1317.9	13.5	1090.7	69.6	1505.8
**XLOC_005914**	NR3C1	"nuclear receptor subfamily 3, group C, member 1 (glucocorticoid receptor)"	9.8	22.9	9.2	25.4	12.9	24.5
**XLOC_007458**	PGR	progesterone receptor	11.6	26.5	10.4	30.9	20.9	30.0
**XLOC_010065**	NR2F2	"nuclear receptor subfamily 2, group F, member 2"	10.5	97.9	6.7	106.5	15.9	119.6
**XLOC_012119**	PAQR8	progestin and adipoQ receptor family member VIII	0.4	1.1	0.4	1.5	0.5	1.5
**XLOC_014279**	PPARG	peroxisome proliferator-activated receptor gamma	4.9	13.3	4.0	25.5	9.4	23.1
**XLOC_016054**	LOC106041605	pre-B-cell leukemia transcription factor 1	7.8	23.6	8.1	30.1	9.7	27.0
**XLOC_016464**	NR5A2	"nuclear receptor subfamily 5, group A, member 2"	0.2	1.5	0.1	25.9	0.3	8.8
**XLOC_017990**	NR0B1	"nuclear receptor subfamily 0, group B, member 1"	59.2	142.2	49.3	139.9	71.1	125.1
**XLOC_019017**	RXRA	"retinoid X receptor, alpha"	1.2	7.0	1.1	7.7	1.2	6.1
**XLOC_019156**	LOC106044184	"cholesterol side-chain cleavage enzyme, mitochondrial"	15.6	442.1	5.1	645.1	18.3	474.2
**XLOC_019349**	STAR	steroidogenic acute regulatory protein	22.6	167.8	16.4	156.2	53.3	203.8
**XLOC_020869**	LOC106045741	3 beta-hydroxysteroid dehydrogenaseDelta 5--4-isomerase-like	12.9	91.1	9.5	304.2	23.8	125.0
**XLOC_022900**	NSDHL	NAD(P) dependent steroid dehydrogenase-like	68.1	34.3	80.9	35.4	65.9	31.3
**XLOC_023486**	LOC106047991	signal transducer and activator of transcription 5A-like	13.4	26.4	11.6	32.1	10.8	27.5

Table S7 Differentially expressed transcription factors during the period between egg-laying and broody goose follicles

gene_id			LSWF	BSWF	LLWF	BLWF	LSYF	BSYF
**XLOC_000018**	TLX1	T-cell leukemia homeobox 1	0.3	0.1	0.4	0.0	0.1	0.0
**XLOC_000056**	VENTX	VENT homeobox	0.8	0.5	1.3	0.5	1.0	0.3
**XLOC_000068**	HHEX	hematopoietically expressed homeobox	1.3	6.1	0.5	5.8	1.7	6.1
**XLOC_000103**	DRGX	dorsal root ganglia homeobox	0.1	1.5	0.7	3.2	7.9	5.3
**XLOC_000123**	PAX2	paired box 2	0.0	0.0			0.0	0.0
**XLOC_000127**	PITX3	paired-like homeodomain 3	0.4	0.1	0.9	0.3	0.2	0.1
**XLOC_000357**	MAPK8	mitogen-activated protein kinase 8	15.0	8.6	14.8	8.6	14.9	8.2
**XLOC_000382**	PITX3	paired-like homeodomain 3	0.5	0.2	1.2	0.4	0.1	0.1
**XLOC_000395**	ADAM8	ADAM metallopeptidase domain 8	2.8	3.0	0.3	2.4	0.6	2.5
**XLOC_000459**	SIRT1	sirtuin 1	24.2	18.8	16.6	18.0	20.0	18.9
**XLOC_000477**	ARID5B	AT rich interactive domain 5B (MRF1-like)	6.5	8.9	5.6	9.2	3.8	7.5
**XLOC_000554**	KAT6B	K(lysine) acetyltransferase 6B	8.7	5.7	11.0	8.5	12.4	5.6
**XLOC_000574**	FOXI3	forkhead box I3	0.3	0.0	0.0	0.0	0.1	0.0
**XLOC_000616**	HTT	huntingtin	9.1	8.4	11.9	8.4	12.0	8.2
**XLOC_000708**	RBPJ	recombination signal binding protein for immunoglobulin kappa J region	15.6	19.2	21.0	25.9	69.2	28.3
**XLOC_000749**	GSC	goosecoid homeobox	0.1	0.8	0.1	0.8	0.2	0.6
**XLOC_000790**	FOXN3	forkhead box N3	14.0	12.3	10.2	12.4	15.4	12.2
**XLOC_000835**	CTBP1	C-terminal binding protein 1	65.3	151.7	80.6	207.1	85.8	218.2
**XLOC_000888**	WFS1	Wolfram syndrome 1 (wolframin)	72.1	46.8	83.2	47.8	81.7	36.0
**XLOC_000892**	MSX1	msh homeobox 1	14.6	7.6	19.6	20.9	21.6	24.4
**XLOC_000944**	PPARGC1A	"peroxisome proliferator-activated receptor gamma, coactivator 1 alpha"	2.7	0.8	2.7	0.9	2.0	0.5

**XLOC_000962**	YY1	YY1 transcription factor	11.1	21.8	12.9	19.8	24.2	22.7
**XLOC_001037**	LOC106037022	glycogen synthase kinase-3 beta-like	153.3	26.6	42.6	41.4	35.2	37.9
**XLOC_001197**	POU1F1	POU class 1 homeobox 1	0.0	0.0	0.1	0.0		
**XLOC_001277**	LOC106037987	group 10 secretory phospholipase A2-like	0.3	0.0	0.0	0.0	0.0	0.0
**XLOC_001290**	BLZF1	basic leucine zipper nuclear factor 1	126.1	51.0	98.9	53.0	82.3	56.4
**XLOC_001356**	LOC106039760	"POU domain, class 2, transcription factor 1"	5.1	2.6	7.5	3.1	4.8	2.0
**XLOC_001462**	TSHZ2	teashirt zinc finger homeobox 2	6.2	6.3	7.2	9.1	9.8	6.4
**XLOC_001483**	TFAP2C	transcription factor AP-2 gamma (activating enhancer binding protein 2 gamma)	0.5	0.2	0.2	0.2	0.1	0.1
**XLOC_001579**	CREB3L1	cAMP responsive element binding protein 3-like 1	6.2	44.9	12.0	55.2	16.0	53.6
**XLOC_001587**	NR1H3	"nuclear receptor subfamily 1, group H, member 3"	24.9	68.9	17.6	72.8	23.8	70.2
**XLOC_001632**	ELF5	E74-like factor 5 (ets domain transcription factor)	0.0	0.0	0.1	0.0	0.1	0.0
**XLOC_001666**	RBPJL	recombination signal binding protein for immunoglobulin kappa J region-like	0.3	0.9	0.1	1.4	0.9	1.2
**XLOC_001675**	HNF4A	"hepatocyte nuclear factor 4, alpha"	0.0	0.1	0.0	0.1	0.0	0.1
**XLOC_001711**	ADNP	activity-dependent neuroprotector homeobox	28.4	25.7	23.6	26.1	25.2	25.3
**XLOC_001720**	SALL4	spalt-like transcription factor 4	19.0	4.7	20.4	8.4	6.7	2.7
**XLOC_001731**	ZNF217	zinc finger protein 217	17.6	16.7	24.4	17.9	24.9	13.4
**XLOC_001800**	MGA	"MGA, MAX dimerization protein"	3.7	4.3	2.6	3.9	3.1	3.4
**XLOC_001838**	SPI1	Spi-1 proto-oncogene	14.3	14.5	5.1	22.0	8.0	18.7
**XLOC_001961**	PHF5A	PHD finger protein 5A	134.6	70.9	132.7	71.6	114.0	85.9
**XLOC_001981**	E2F7	E2F transcription factor 7	3.0	1.5	2.6	1.6	1.8	1.7
**XLOC_002064**	DBX2	developing brain homeobox 2	0.3	0.2	0.4	0.1	0.4	0.2
**XLOC_002139**	TEF	thyrotrophic embryonic factor	12.1	31.4	12.6	26.7	17.8	34.5
**XLOC_002186**	YEATS4	YEATS domain containing 4	135.2	53.2	89.7	61.0	77.6	64.2
**XLOC_002205**	HMGA2	high mobility group AT-hook 2	3.7	2.7	6.3	2.6	19.2	2.6
**XLOC_002323**	ZBTB18	zinc finger and BTB domain containing 18	4.0	5.3	3.4	6.5	3.8	5.1
**XLOC_002407**	FOSL2	FOS-like antigen 2	6.8	12.2	3.2	13.6	4.0	9.6

**XLOC_002424**	SIX2	SIX homeobox 2	0.1	0.2	0.0	0.3	0.0	0.4
**XLOC_002432**	EGLN1	egl-9 family hypoxia-inducible factor 1	18.7	38.0	19.9	40.5	30.2	40.4
**XLOC_002533**	NFKBIE	"nuclear factor of kappa light polypeptide gene enhancer in B-cells inhibitor, epsilon"	8.0	13.2	4.7	14.4	3.2	13.5
**XLOC_002573**	EPAS1	endothelial PAS domain protein 1	58.3	66.9	67.0	60.8	49.2	66.8
**XLOC_002584**	GLI2	GLI family zinc finger 2	2.6	10.6	1.7	9.6	1.7	10.1
**XLOC_002624**	TBR1	"T-box, brain, 1"	1.3	0.7	2.6	0.7	0.5	0.2
**XLOC_002637**	CSRNP3	cysteine-serine-rich nuclear protein 3	1.7	0.4	1.8	0.6	1.5	0.3
**XLOC_002647**	CERS6	ceramide synthase 6	1.3	1.8	1.2	1.9	1.3	2.2
**XLOC_002672**	DLX1	distal-less homeobox 1	0.4	0.4	0.1	0.2	0.0	0.2
	HOXD12	homeobox D12			0.0	0.0		
**XLOC_002690**	HOXD11	homeobox D11	0.0	0.5	0.0	0.2	2.0	0.6
**XLOC_002693**	HOXD9	homeobox D9	1.0	1.0	0.4	2.3	1.7	3.4
**XLOC_002694**	HOXD8	homeobox D8	1.0	2.3	0.1	2.4	0.9	2.8
**XLOC_002695**	HOXD4	homeobox D4	0.1	0.3	0.0	0.0	0.1	0.1
**XLOC_002741**	CREB1	cAMP responsive element binding protein 1	5.0	7.7	3.6	8.6	7.4	9.5
**XLOC_002767**	TFCP2L1	transcription factor CP2-like 1	5.7	3.3	7.2	3.3	5.9	1.5
**XLOC_002881**	NFE2L2	"nuclear factor, erythroid 2-like 2"	44.2	47.4	32.5	55.0	39.5	49.9
**XLOC_002913**	KLF7	Kruppel-like factor 7 (ubiquitous)	2.1	2.1	1.5	1.9	2.8	2.3
**XLOC_002931**	DIP2A	disco-interacting protein 2 homolog A	11.6	12.7	11.0	12.2	13.9	12.7
**XLOC_002942**	HDAC9	histone deacetylase 9	3.7	4.9	4.7	5.0	7.2	4.1
**XLOC_002986**	CREB5	cAMP responsive element binding protein 5	0.6	0.8	1.3	0.6	1.0	0.5
**XLOC_003004**	KAT2B	K(lysine) acetyltransferase 2B	5.9	10.9	6.9	12.2	7.5	13.0
**XLOC_003015**	RARB	"retinoic acid receptor, beta"	19.4	25.2	12.7	32.5	9.3	24.8
**XLOC_003058**	TWIST1	twist family bHLH transcription factor 1	0.3	3.1	0.0	5.8	0.2	2.0
**XLOC_003101**	HOXA1	homeobox A1	14.5	6.5	15.9	4.6	9.2	3.7
**XLOC_003102**	HOXA2	homeobox A2	0.3	0.6	0.1	0.6	0.1	0.5

**XLOC_003104**	HOXA4	homeobox A4	3.8	3.6	3.8	2.5	3.1	2.4
**XLOC_003105**	HOXA9	homeobox A9	2.1	4.2	1.6	2.9	6.3	4.1
**XLOC_003106**	HOXA10	homeobox A10	5.3	1.6	0.5	0.1	8.9	3.5
**XLOC_003107**	HOXA11	homeobox A11	0.0	0.0			0.3	0.0
**XLOC_003149**	THRB	"thyroid hormone receptor, beta"	0.7	2.0	0.2	2.9	0.5	2.1
**XLOC_003151**	THRB	"thyroid hormone receptor, beta"	1.5	0.1	1.7	0.0	0.4	0.1
**XLOC_003170**	EOMES	eomesodermin	33.2	10.4	39.7	15.6	15.5	6.5
**XLOC_003267**	GCM1	glial cells missing homolog 1 (Drosophila)	0.0	0.0	0.0	0.0	0.1	0.1
**XLOC_003429**	NBN	nibrin	13.9	9.3	16.4	9.2	12.9	8.9
**XLOC_003443**	E2F5	"E2F transcription factor 5, p130-binding"	96.7	169.3	152.5	199.6	324.5	249.0
**XLOC_003481**	MSC	musculin	1.6	1.5	0.4	1.7	0.8	2.1
**XLOC_003498**	COPS5	COP9 signalosome subunit 5	185.9	91.2	138.4	106.7	180.5	108.7
**XLOC_003563**	E2F5	"E2F transcription factor 5, p130-binding"	47.3	36.3	63.2	28.7	65.1	26.1
**XLOC_003590**	ZFHX4	zinc finger homeobox 4	0.6	1.1	0.5	0.9	0.6	0.9
**XLOC_003594**	HNF4G	"hepatocyte nuclear factor 4, gamma"	0.0	0.0	0.1	0.2	0.1	0.3
**XLOC_003683**	MYCN	v-myc avian myelocytomatosis viral oncogene neuroblastoma derived homolog	5.5	3.3	1.9	4.6	2.7	4.4
**XLOC_003723**	GSX2	GS homeobox 2	1.1	0.6	1.1	0.6	0.3	0.3
**XLOC_003741**	MYT1L	myelin transcription factor 1-like	0.1	0.1	0.1	0.0	0.0	0.0
**XLOC_003768**	KLF11	Kruppel-like factor 11	2.6	24.3	0.9	25.0	2.9	22.7
**XLOC_003778**	E2F6	E2F transcription factor 6	20.8	7.9	16.1	6.6	15.7	5.7
**XLOC_003817**	NFXL1	"nuclear transcription factor, X-box binding-like 1"	106.6	60.5	134.2	33.9	154.6	46.7
**XLOC_003836**	CLOCK	clock circadian regulator	4.9	5.4	3.3	6.0	4.4	6.0
**XLOC_003842**	ETV6	ets variant 6	13.8	16.9	10.3	18.6	11.7	18.7
**XLOC_003846**	CREBL2	cAMP responsive element binding protein-like 2	47.6	36.4	44.1	35.2	37.6	28.7
**XLOC_003887**	KDM5A	lysine (K)-specific demethylase 5A	57.5	18.5	40.3	18.7	24.5	15.2
**XLOC_003940**	SPIC	Spi-C transcription factor (Spi-1PU.1 related)	0.1	0.0	0.0	0.1	0.1	0.0

**XLOC_003947**	LRP6	low density lipoprotein receptor-related protein 6	3.2	5.8	2.5	5.3	4.1	5.3
**XLOC_004020**	LOC106030952	zinc finger protein 501-like	1.2	0.6	0.6	0.9	0.4	0.7
**XLOC_004049**	CREB3L2	cAMP responsive element binding protein 3-like 2	41.8	43.9	43.0	52.6	56.4	45.1
**XLOC_004073**	GNPTAB	"N-acetylglucosamine-1-phosphate transferase, alpha and beta subunits"	26.2	24.6	27.7	28.8	27.8	28.5
**XLOC_004094**	FOXN4	forkhead box N4	1.4	0.9	1.5	0.7	0.6	1.1
**XLOC_004105**	CMKLR1	chemerin chemokine-like receptor 1	0.4	2.0	0.2	1.9	0.2	1.8
**XLOC_004174**	PUS1	pseudouridylate synthase 1	21.3	22.0	26.9	23.4	22.3	24.2
**XLOC_004227**	LOC106031301	T-box-containing protein TBX6L	2.4	2.0	2.8	1.0	2.3	1.3
**XLOC_004244**	HNF1A	HNF1 homeobox A	1.3	1.1	1.0	0.8	1.5	0.6
**XLOC_004245**	HNF1A	HNF1 homeobox A	0.4	0.9	0.7	0.5	1.4	0.4
**XLOC_004258**	PLA2G1B	"phospholipase A2, group IB (pancreas)"	0.6	0.1	0.2	0.1	0.1	0.0
**XLOC_004328**	CUX2	cut-like homeobox 2	2.3	2.7	1.6	2.6	1.0	2.7
**XLOC_004338**	PSMD9	"proteasome (prosome, macropain) 26S subunit, non-ATPase, 9"	42.0	37.7	39.8	38.7	29.6	41.8
**XLOC_004409**	TBX1	T-box 1	1.3	1.3	1.4	1.8	0.8	1.2
**XLOC_004456**	HNF1A	HNF1 homeobox A	0.3	0.1	0.2	0.2	0.3	0.2
	LHX5	LIM homeobox 5			0.1	0.0	0.0	0.1
**XLOC_004496**	TBX5	T-box 5	4.8	1.1	3.2	0.9	0.8	0.5
**XLOC_004497**	TBX3	T-box 3	2.9	19.7	1.5	16.7	2.1	17.7
**XLOC_004535**	TRERF1	transcriptional regulating factor 1	38.3	18.8	25.5	18.7	19.1	18.9
**XLOC_004549**	TRAF5	TNF receptor-associated factor 5	1.7	1.8	1.0	2.5	1.4	2.4
**XLOC_004634**	MTA3	"metastasis associated 1 family, member 3"	25.9	15.3	30.3	11.7	25.5	13.0
**XLOC_004699**	CENPF	"centromere protein F, 350400kDa"	20.7	5.1	14.4	4.1	14.5	7.2
**XLOC_004707**	ATF3	activating transcription factor 3	6.9	6.1	8.9	7.1	8.0	6.3
**XLOC_004735**	ZFP36L1	ZFP36 ring finger protein-like 1	117.5	180.8	46.8	209.6	36.3	165.8
**XLOC_004757**	MEIS2	Meis homeobox 2	11.8	32.5	2.6	54.3	4.4	41.9
**XLOC_004784**	FOXG1	forkhead box G1	0.3	0.0	0.3	0.1	0.1	0.1

**XLOC_004871**	PRKD1	protein kinase D1	4.2	6.7	3.9	7.1	6.9	7.7
**XLOC_004874**	STRN3	"striatin, calmodulin binding protein 3"	52.9	32.3	36.9	37.8	33.6	40.2
**XLOC_004890**	IRF7	interferon regulatory factor 7	2.2	6.0	1.2	7.0	1.3	6.9
**XLOC_004933**	SUFU	suppressor of fused homolog (Drosophila)	29.6	28.8	39.0	35.4	31.5	31.5
**XLOC_004942**	TAF5	"TAF5 RNA polymerase II, TATA box binding protein (TBP)-associated factor, 100kDa"	13.0	11.9	12.9	12.0	12.6	12.9
**XLOC_004943**	PDCD11	programmed cell death 11	17.1	10.9	21.1	12.3	17.4	13.0
**XLOC_005051**	PCGF6	polycomb group ring finger 6	6.5	6.3	6.0	5.8	10.0	5.9
**XLOC_005137**	ZNF639	zinc finger protein 639	77.0	21.4	31.8	22.3	42.6	23.5
**XLOC_005187**	FOXK1	forkhead box K1	12.6	8.5	9.7	9.2	5.3	7.9
**XLOC_005223**	LOC106032117	chemokine-like receptor 1	1.9	1.3	2.5	1.0	1.2	0.7
**XLOC_005331**	CARD11	"caspase recruitment domain family, member 11"	1.6	1.8	0.9	2.2	1.1	1.9
**XLOC_005407**	SMAD1	SMAD family member 1	15.4	32.4	15.7	39.3	21.0	32.2
**XLOC_005412**	POU4F2	POU class 4 homeobox 2	1.2	0.2	1.2	0.1	0.7	0.5
**XLOC_005510**	NR3C2	"nuclear receptor subfamily 3, group C, member 2"	10.5	6.1	12.1	5.5	8.9	4.5
**XLOC_005584**	HSF2	heat shock transcription factor 2	49.8	41.0	55.8	37.0	60.3	39.5
**XLOC_005601**	HEY2	hes-related family bHLH transcription factor with YRPW motif 2	46.3	20.4	58.4	16.7	46.6	16.4
**XLOC_005638**	TCF21	transcription factor 21	2.1	158.4	1.3	191.6	4.7	221.5
**XLOC_005651**	TNFAIP3	"tumor necrosis factor, alpha-induced protein 3"	15.4	7.0	14.8	7.0	8.4	6.0
**XLOC_005746**	CITED2	"Cbpp300-interacting transactivator, with GluAsp-rich carboxy-terminal domain, 2"	4.5	12.4	3.4	11.9	6.3	12.4
**XLOC_005756**	HDAC2	histone deacetylase 2	68.8	52.7	81.0	60.6	93.6	64.1
**XLOC_005817**	SIM1	single-minded family bHLH transcription factor 1	4.1	1.2	6.1	1.3	3.5	0.5
**XLOC_005854**	GTF3C6	"general transcription factor IIIC, polypeptide 6, alpha 35kDa"	14.1	10.0	15.3	9.4	10.2	7.8
**XLOC_005867**	FOXO3	forkhead box O3	7.4	23.3	4.6	29.7	4.2	18.4
**XLOC_005870**	NR2E1	"nuclear receptor subfamily 2, group E, member 1"	52.0	51.1	71.9	43.3	83.8	50.1
**XLOC_005877**	PRDM1	"PR domain containing 1, with ZNF domain"	1.7	1.3	0.8	0.9	0.5	0.9
**XLOC_005893**	POU3F2	POU class 3 homeobox 2	0.6	0.0	0.3	0.2	0.2	0.3

**XLOC_005914**	NR3C1	"nuclear receptor subfamily 3, group C, member 1 (glucocorticoid receptor)"	9.8	22.9	9.2	25.4	12.9	24.5
**XLOC_005920**	AFF4	"AF4FMR2 family, member 4"	5.0	8.3	3.9	9.7	5.8	9.5
**XLOC_005926**	IRF1	interferon regulatory factor 1	17.0	24.8	17.7	30.4	8.9	24.7
**XLOC_005940**	TCF7	"transcription factor 7 (T-cell specific, HMG-box)"	0.7	0.5	0.2	0.6	0.4	0.3
**XLOC_005955**	SMAD5	SMAD family member 5	12.2	26.4	17.7	28.0	39.8	30.5
**XLOC_006013**	CNOT8	"CCR4-NOT transcription complex, subunit 8"	65.7	46.3	65.0	47.4	63.4	54.3
	PITX1	paired-like homeodomain 1			0.0	0.0	0.0	0.0
**XLOC_006219**	PPAP2B	phosphatidic acid phosphatase type 2B	6.3	14.1	2.1	18.2	3.4	14.6
**XLOC_006272**	DMBX1	diencephalonmesencephalon homeobox 1	1.2	0.4	2.0	0.5	0.7	0.2
**XLOC_006279**	LOC106032954	phosphatidylinositol 3-kinase regulatory subunit alpha-like	0.0	0.1	0.0	0.1		
**XLOC_006315**	NFIA	nuclear factor IA	5.6	16.1	2.2	13.1	4.4	15.3
**XLOC_006333**	DMRTB1	"DMRT-like family B with proline-rich C-terminal, 1"	0.1	0.0	0.1	0.1	0.1	0.1
**XLOC_006466**	CEBPG	"CCAATenhancer binding protein (CEBP), gamma"	40.5	36.9	50.1	35.0	69.4	42.6
**XLOC_006526**	SETD6	SET domain containing 6	10.8	8.4	15.6	7.6	10.0	8.0
**XLOC_006547**	E2F4	"E2F transcription factor 4, p107p130-binding"	16.0	11.4	13.1	13.0	7.9	11.9
**XLOC_006593**	LOC106033227	transcription initiation factor TFIID subunit 4-like	3.0	2.0	1.9	2.0	1.2	1.6
**XLOC_006620**	MAF	v-maf avian musculoaponeurotic fibrosarcoma oncogene homolog	7.3	11.4	2.7	17.3	5.9	10.1
**XLOC_006659**	TFAP4	transcription factor AP-4 (activating enhancer binding protein 4)	8.1	9.9	9.4	9.3	8.8	13.0
**XLOC_006664**	CREBBP	CREB binding protein	6.7	10.2	5.2	10.1	5.2	9.6
**XLOC_006745**	LOC106033331	forkhead box protein I1c	0.3	2.5	0.1	1.8	0.8	2.7
**XLOC_006791**	NLRC3	"NLR family, CARD domain containing 3"	0.2	0.7	0.1	0.6	0.1	0.8
**XLOC_006814**	GTF3C1	"general transcription factor IIIC, polypeptide 1, alpha 220kDa"	26.4	13.2	20.5	13.3	13.4	13.2
**XLOC_006943**	TCF12	transcription factor 12	43.7	62.0	40.0	71.8	51.2	73.9
**XLOC_006970**	FOXB1	forkhead box B1	2.4	0.3	1.2	0.3	0.3	0.2
**XLOC_007031**	ONECUT1	one cut homeobox 1	0.3	0.1	0.4	0.2	0.2	0.1
**XLOC_007083**	RORA	RAR-related orphan receptor A	1.8	1.7	1.8	1.7	2.7	1.6

**XLOC_007204**	DBX1	developing brain homeobox 1	0.4	0.0	0.6	0.2	0.3	0.1
**XLOC_007237**	PAX6	paired box 6	0.1	0.2	0.3	0.1	0.1	0.2
**XLOC_007246**	WT1	Wilms tumor 1	536.9	102.9	171.4	98.0	135.1	89.6
**XLOC_007406**	BCL10	B-cell CLLlymphoma 10	4.7	11.7	4.9	13.5	9.1	15.1
**XLOC_007458**	PGR	progesterone receptor	11.6	26.5	10.4	30.9	20.9	30.0
**XLOC_007538**	MED17	mediator complex subunit 17	95.6	25.7	55.8	25.0	42.7	23.3
**XLOC_007571**	TAB2	TGF-beta activated kinase 1MAP3K7 binding protein 2	19.6	15.4	18.5	16.8	16.0	15.9
**XLOC_007582**	ESR1	estrogen receptor 1	1.9	4.0	1.6	4.2	3.2	4.5
**XLOC_007731**	HDAC3	histone deacetylase 3	39.9	38.7	45.5	36.4	28.4	38.3
**XLOC_007755**	FOXI1	forkhead box I1	0.2	0.1	0.2	0.2	0.1	0.2
**XLOC_007793**	LOC106034406	uncharacterized LOC106034406	0.0	2.4	0.0	2.4	0.1	2.5
**XLOC_007910**	GBX2	gastrulation brain homeobox 2	6.3	1.6	5.9	0.7	1.7	0.5
**XLOC_007993**	BPTF	bromodomain PHD finger transcription factor	11.2	7.1	9.8	7.5	9.3	6.8
**XLOC_008008**	TRIM25	tripartite motif containing 25	4.4	8.5	2.9	15.8	7.3	14.7
**XLOC_008066**	SPHK1	sphingosine kinase 1	12.5	16.1	6.5	13.0	3.6	16.7
**XLOC_008123**	ZNF207	zinc finger protein 207	19.0	38.6	30.6	36.8	33.1	43.3
**XLOC_008138**	HLF	hepatic leukemia factor	10.7	7.3	14.8	6.0	11.8	5.2
**XLOC_008204**	FOXJ1	forkhead box J1	17.6	5.9	19.7	6.1	7.2	2.6
**XLOC_008228**	TFAP2A	transcription factor AP-2 alpha (activating enhancer binding protein 2 alpha)	2.0	0.5	1.0	0.3	0.4	0.4
**XLOC_008321**	TGIF1	TGFB-induced factor homeobox 1	5.7	11.8	5.4	11.1	6.4	8.6
**XLOC_008461**	TSC22D1	"TSC22 domain family, member 1"	9.6	8.2	10.2	7.4	7.9	6.4
**XLOC_008472**	ELF1	E74-like factor 1 (ets domain transcription factor)	20.7	18.3	11.3	21.3	18.7	21.4
**XLOC_008526**	GTF2F2	"general transcription factor IIF, polypeptide 2, 30kDa"	26.3	15.3	19.2	18.5	21.8	21.6
**XLOC_008534**	TNFSF11	"tumor necrosis factor (ligand) superfamily, member 11"	25.4	3.3	12.1	2.3	5.5	2.2
**XLOC_008539**	NAA16	"N(alpha)-acetyltransferase 16, NatA auxiliary subunit"	4.2	5.6	6.6	5.3	8.9	5.8
**XLOC_008604**	IRF2	interferon regulatory factor 2	9.0	14.4	7.2	15.2	8.9	14.6

**XLOC_008698**	MTDH	metadherin	40.0	45.6	34.4	51.6	42.0	57.9
**XLOC_008727**	ZFPM2	"zinc finger protein, FOG family member 2"	39.5	24.4	31.9	29.0	28.9	33.5
**XLOC_008765**	RNF19A	"ring finger protein 19A, RBR E3 ubiquitin protein ligase"	69.8	30.3	43.6	29.8	37.8	26.3
**XLOC_008780**	KLF10	Kruppel-like factor 10	31.8	15.6	18.4	20.5	9.0	14.9
**XLOC_008792**	ABRA	actin binding Rho activating protein	0.0	0.1	0.0	0.1	0.1	0.0
**XLOC_008880**	TBX18	T-box 18	0.3	0.2	0.2	0.1	0.4	0.3
**XLOC_008907**	BACH2	"BTB and CNC homology 1, basic leucine zipper transcription factor 2"	2.0	2.1	1.2	1.9	1.7	1.6
**XLOC_008966**	MITF	microphthalmia-associated transcription factor	9.0	5.5	5.9	6.3	3.3	5.2
**XLOC_009027**	FOXP1	forkhead box P1	5.5	9.6	3.6	11.0	7.4	9.8
**XLOC_009265**	SHOX	short stature homeobox	0.2	0.0	0.2	0.0	0.1	0.0
**XLOC_009396**	ISX	intestine-specific homeobox	0.0	0.0	0.0	0.1	0.0	0.2
**XLOC_009440**	ATF4	activating transcription factor 4	144.2	317.7	159.2	335.8	152.3	354.7
**XLOC_009454**	EP300	E1A binding protein p300	29.6	11.5	33.2	14.6	26.5	13.9
**XLOC_009455**	LOC106035824	histone acetyltransferase p300-like	148.4	15.4	205.0	43.9	185.5	46.5
**XLOC_009495**	RBFOX2	"RNA binding protein, fox-1 homolog (C. elegans) 2"	10.9	30.3	5.2	35.0	7.5	31.3
**XLOC_009546**	IRX1	iroquois homeobox 1	15.3	0.5	4.0	0.1	1.5	0.2
**XLOC_009591**	LOC106035954	iroquois-class homeodomain protein irx-2-like	0.5	0.2	0.3	0.1	0.8	0.1
**XLOC_009627**	ZNF236	zinc finger protein 236	1.2	3.4	1.5	3.1	3.7	3.4
**XLOC_009631**	TSHZ1	teashirt zinc finger homeobox 1	3.5	5.9	4.1	6.6	4.5	4.8
**XLOC_009685**	LOC106036141	histone H4 transcription factor-like	3.0	1.2	4.1	0.7	2.5	0.4
**XLOC_009696**	ERG	v-ets avian erythroblastosis virus E26 oncogene homolog	2.1	10.0	1.0	10.9	1.8	10.7
**XLOC_009703**	RIPPLY3	ripply transcriptional repressor 3	0.0	0.1	0.1	0.4	0.2	0.2
**XLOC_009734**	PKNOX1	PBXknotted 1 homeobox 1	15.2	12.1	13.7	12.8	14.4	12.7
**XLOC_009771**	RIPPLY3	ripply transcriptional repressor 3	0.1	0.1	0.0	0.1	0.1	0.1
**XLOC_009772**	SIM2	single-minded family bHLH transcription factor 2	0.6	0.2	0.5	0.0	0.4	0.1
**XLOC_009790**	LOC106036243	phospholipid scramblase 1-like	28.6	26.9	28.8	28.7	28.8	27.3

**XLOC_009801**	TFDP2	transcription factor Dp-2 (E2F dimerization partner 2)	12.8	12.5	13.6	14.7	17.1	17.1
**XLOC_009838**	PAX3	paired box 3	1.1	0.3	1.2	0.4	0.6	0.2
**XLOC_009865**	ZBTB38	zinc finger and BTB domain containing 38	6.6	3.7	6.7	4.1	6.7	3.8
**XLOC_009957**	CREB3	cAMP responsive element binding protein 3	31.2	36.0	38.0	33.0	20.7	28.2
**XLOC_009972**	ZNF131	zinc finger protein 131	12.0	5.4	13.3	7.2	17.0	6.4
**XLOC_010065**	NR2F2	"nuclear receptor subfamily 2, group F, member 2"	10.6	97.9	6.7	106.6	15.9	119.6
**XLOC_010077**	MEF2A	myocyte enhancer factor 2A	28.1	15.3	11.6	19.1	11.6	18.5
**XLOC_010234**	DAP	death-associated protein	96.9	153.0	144.3	168.6	407.5	195.6
**XLOC_010337**	REL	v-rel avian reticuloendotheliosis viral oncogene homolog	6.1	7.5	5.6	9.0	6.1	7.9
**XLOC_010353**	NR2C2	"nuclear receptor subfamily 2, group C, member 2"	5.4	9.4	7.4	9.7	13.4	10.9
**XLOC_010427**	IRAK2	interleukin-1 receptor-associated kinase 2	19.9	15.9	17.9	18.5	21.7	17.5
**XLOC_010526**	LOC106036866	zinc finger protein 260-like	0.2	1.0	0.1	1.2	0.1	1.1
**XLOC_010541**	CSRNP1	cysteine-serine-rich nuclear protein 1	10.4	17.6	7.4	21.6	7.9	18.2
**XLOC_010640**	ELK3	"ELK3, ETS-domain protein (SRF accessory protein 2)"	7.8	32.9	3.5	42.5	10.0	33.9
**XLOC_010664**	NR1H4	"nuclear receptor subfamily 1, group H, member 4"	0.2	0.0	0.5	0.1	0.5	0.1
**XLOC_010702**	NR2C1	"nuclear receptor subfamily 2, group C, member 1"	16.8	23.3	20.4	19.3	31.1	20.7
**XLOC_010761**	GABPA	"GA binding protein transcription factor, alpha subunit 60kDa"	36.7	23.7	20.6	24.6	23.4	28.7
**XLOC_010860**	TRAF2	TNF receptor-associated factor 2	8.8	13.2	11.7	14.7	7.5	13.2
**XLOC_010897**	TRIM32	tripartite motif containing 32	1.7	3.3	2.7	3.0	2.9	3.1
**XLOC_010956**	ARID4A	AT rich interactive domain 4A (RBP1-like)	11.8	8.1	4.4	9.3	5.3	9.0
**XLOC_010972**	SIX6	SIX homeobox 6	0.0	0.0	0.0	0.0		
**XLOC_010981**	HIF1A	"hypoxia inducible factor 1, alpha subunit (basic helix-loop-helix transcription factor)"	13.6	29.4	14.2	33.0	23.4	29.7
**XLOC_011020**	AKT1	v-akt murine thymoma viral oncogene homolog 1	21.5	32.7	20.1	43.0	16.7	36.6
**XLOC_011046**	LOC106037285	"chromosome unknown open reading frame, human C14orf39"	0.1	0.0	0.2	0.0	0.1	0.0
**XLOC_011062**	ESR2	estrogen receptor 2 (ER beta)	1.3	1.8	1.4	1.0	1.0	1.6
**XLOC_011167**	SMAD2	SMAD family member 2	110.3	127.1	122.1	247.3	576.4	264.1

**XLOC_011174**	SMAD7	SMAD family member 7	3.9	11.0	3.4	9.1	12.3	11.9
**XLOC_011254**	FOXO4	forkhead box O4	10.9	22.6	19.1	21.4	16.4	18.9
**XLOC_011279**	ZIC3	Zic family member 3	0.3	0.0	0.1	0.0	0.0	0.2
**XLOC_011331**	MEOX2	mesenchyme homeobox 2	0.5	2.3	0.2	2.0	0.5	2.4
**XLOC_011335**	ETV1	ets variant 1	11.8	11.2	12.9	9.4	14.2	10.4
**XLOC_011347**	DLX5	distal-less homeobox 5	0.1	0.3	0.0	0.1	0.2	0.2
**XLOC_011388**	DLX6	distal-less homeobox 6	0.6	0.2	0.6	0.2	0.1	0.1
**XLOC_011422**	TAF4B	"TAF4b RNA polymerase II, TATA box binding protein (TBP)-associated factor, 105kDa"	163.8	14.3	58.1	8.5	34.7	8.9
**XLOC_011560**	TAF7L	"TAF7-like RNA polymerase II, TATA box binding protein (TBP)-associated factor, 50kDa"	200.9	76.0	188.4	68.2	85.0	61.6
**XLOC_011597**	PASD1	PAS domain containing 1	1.2	0.8	1.2	1.0	0.8	0.9
**XLOC_011653**	TAF1	"TAF1 RNA polymerase II, TATA box binding protein (TBP)-associated factor, 250kDa"	27.4	25.1	30.2	26.6	28.5	25.4
**XLOC_011724**	SCML2	sex comb on midleg-like 2 (Drosophila)	1.5	3.2	2.3	2.8	3.6	3.3
**XLOC_011761**	RUNX3	runt-related transcription factor 3	18.1	3.1	10.5	2.7	2.0	3.1
**XLOC_011774**	MYCL	v-myc avian myelocytomatosis viral oncogene lung carcinoma derived homolog	27.1	14.1	23.9	11.2	19.5	11.9
**XLOC_011800**	HDAC1	histone deacetylase 1	17.1	18.2	17.2	18.5	17.3	19.5
	LOC106038051	transcription factor AP-2-epsilon-like			0.0	0.1		
**XLOC_011827**	LOC106038050	transcription factor AP-2-epsilon-like	0.0	0.1	0.0	0.1	0.0	0.0
**XLOC_011847**	MTF1	metal-regulatory transcription factor 1	5.7	4.0	7.5	4.5	3.9	2.8
**XLOC_011902**	GRHL3	grainyhead-like 3 (Drosophila)	3.3	0.1	3.2	0.1	2.0	0.2
**XLOC_011969**	MTF1	metal-regulatory transcription factor 1	28.5	7.1	17.9	7.4	11.6	7.2
**XLOC_012036**	HMBOX1	homeobox containing 1	5.6	4.5	3.6	4.2	4.4	4.4
**XLOC_012045**	GATA4	GATA binding protein 4	33.1	74.6	48.9	66.0	81.8	76.1
**XLOC_012070**	RUNX2	runt-related transcription factor 2	0.3	3.4	0.1	4.2	2.7	2.0
**XLOC_012098**	SOX7	SRY (sex determining region Y)-box 7	1.3	6.9	0.5	5.4	0.7	5.9
**XLOC_012124**	TFAP2B	transcription factor AP-2 beta (activating enhancer binding protein 2 beta)	8.2	1.4	7.6	1.7	3.8	0.9
**XLOC_012125**	TFAP2D	transcription factor AP-2 delta (activating enhancer binding protein 2 delta)	0.0	0.0	0.0	0.0	0.0	0.0

**XLOC_012135**	SUPT3H	suppressor of Ty 3 homolog (S. cerevisiae)	8.7	13.6	8.7	16.0	14.9	14.5
**XLOC_012169**	ARNTL2	aryl hydrocarbon receptor nuclear translocator-like 2	7.6	8.9	7.2	9.9	12.7	9.3
**XLOC_012218**	PPARA	peroxisome proliferator-activated receptor alpha	4.8	3.0	5.3	3.1	7.5	3.2
**XLOC_012228**	BHLHE41	"basic helix-loop-helix family, member e41"	31.6	17.8	11.4	24.2	11.6	27.5
**XLOC_012276**	SOX4	SRY (sex determining region Y)-box 4	4.2	25.0	3.5	29.1	13.2	21.5
**XLOC_012289**	NFATC1	"nuclear factor of activated T-cells, cytoplasmic, calcineurin-dependent 1"	2.6	5.3	2.0	7.9	3.0	7.3
**XLOC_012294**	ADNP2	ADNP homeobox 2	17.4	11.6	13.7	11.8	15.1	11.2
**XLOC_012336**	CTNNB1	"catenin (cadherin-associated protein), beta 1, 88kDa"	126.9	225.0	122.1	258.4	160.2	325.3
**XLOC_012408**	ST18	"suppression of tumorigenicity 18, zinc finger"	3.2	1.0	3.2	0.3	1.8	0.3
**XLOC_012432**	SNAI2	snail family zinc finger 2	9.9	152.2	5.5	203.6	19.9	125.2
**XLOC_012437**	ST18	"suppression of tumorigenicity 18, zinc finger"	0.1	0.1	0.1	0.1	0.1	0.0
**XLOC_012473**	ZHX1	zinc fingers and homeoboxes 1	7.6	12.1	6.1	13.1	6.1	13.8
**XLOC_012484**	TAF2	"TAF2 RNA polymerase II, TATA box binding protein (TBP)-associated factor, 150kDa"	48.4	36.0	41.4	40.4	32.2	41.5
**XLOC_012496**	MYC	v-myc avian myelocytomatosis viral oncogene homolog	7.8	25.0	5.7	24.4	10.9	27.1
**XLOC_012517**	ZHX2	zinc fingers and homeoboxes 2	3.5	9.1	3.8	9.6	4.3	8.7
	LOC106038555	homeobox protein CDX-1-like			0.1	0.0		
**XLOC_012537**	LOC106038556	homeobox protein CDX-4-like	0.0	0.0	0.0	0.0		
**XLOC_012564**	PSMD10	"proteasome (prosome, macropain) 26S subunit, non-ATPase, 10"	47.6	35.1	53.7	38.3	46.5	36.5
**XLOC_012588**	RLIM	"ring finger protein, LIM domain interacting"	18.4	12.8	20.3	12.9	18.2	11.8
**XLOC_012624**	LOC106038686	PR domain zinc finger protein 5-like	0.6	3.9	1.0	4.0	2.0	4.3
**XLOC_012650**	PITX2	paired-like homeodomain 2	2.7	0.4	2.5	0.3	0.2	0.0
**XLOC_012661**	LEF1	lymphoid enhancer-binding factor 1	10.6	10.8	4.3	10.9	2.9	13.5
**XLOC_012755**	GATA3	GATA binding protein 3	4.4	3.4	3.7	4.1	1.0	4.0
**XLOC_012835**	VSX2	visual system homeobox 2	0.8	0.1	0.3	0.1	0.3	0.1
**XLOC_012849**	FOS	FBJ murine osteosarcoma viral oncogene homolog	41.7	20.9	47.6	22.5	22.6	15.0
**XLOC_012859**	ESRRB	estrogen-related receptor beta	1.5	1.2	2.0	1.9	3.4	1.5

**XLOC_012898**	FOXA1	forkhead box A1	0.1	0.0	0.1	0.0	0.0	0.0
**XLOC_012988**	DIP2C	disco-interacting protein 2 homolog C	7.5	8.1	6.6	8.8	8.9	9.0
	LOC106038953	homeobox protein HMX3-like			0.1	0.0		
**XLOC_013208**	KLF3	Kruppel-like factor 3 (basic)	11.4	6.5	5.2	6.2	5.9	6.2
**XLOC_013264**	APBB2	"amyloid beta (A4) precursor protein-binding, family B, member 2"	17.7	18.4	11.9	18.8	13.0	19.0
**XLOC_013272**	MAP3K13	mitogen-activated protein kinase kinase kinase 13	13.2	6.8	15.7	6.8	9.6	5.2
**XLOC_013297**	UBXN7	UBX domain protein 7	14.3	8.0	11.6	10.3	9.4	10.3
**XLOC_013314**	LOC106039179	extensin-like	9.4	4.1	4.4	3.4	1.8	2.8
**XLOC_013383**	ETV5	ets variant 5	12.6	20.7	15.6	19.5	7.9	18.7
**XLOC_013453**	FEV	FEV (ETS oncogene family)	10.6	6.1	6.5	2.2	6.0	2.3
**XLOC_013455**	RNF25	ring finger protein 25	11.9	8.7	13.9	9.2	7.5	8.8
	EN1	engrailed homeobox 1			0.2	0.0	0.0	0.2
**XLOC_013505**	STK36	serinethreonine kinase 36	2.3	4.0	1.9	4.8	2.4	4.5
**XLOC_013626**	T	T brachyury transcription factor	5.1	1.3	4.4	1.6	1.7	0.9
**XLOC_013744**	CYLD	cylindromatosis (turban tumor syndrome)	5.5	5.8	5.1	5.9	11.5	6.1
**XLOC_013780**	TSHZ3	teashirt zinc finger homeobox 3	6.3	12.0	6.0	13.4	19.3	14.8
**XLOC_013833**	GATAD1	GATA zinc finger domain containing 1	17.9	18.8	29.5	19.6	30.6	23.3
**XLOC_013905**	TRIM29	tripartite motif containing 29	2.4	0.3	0.6	0.2	0.2	0.3
**XLOC_014016**	POU2F3	POU class 2 homeobox 3	1.8	0.7	3.4	0.6	1.8	0.5
**XLOC_014029**	HINFP	histone H4 transcription factor	24.6	18.8	36.9	17.8	19.9	17.0
**XLOC_014066**	FOXR1	forkhead box R1	0.1	0.3	0.4	0.3	0.8	0.3
**XLOC_014228**	STAT1	"signal transducer and activator of transcription 1, 91kDa"	109.9	146.2	115.7	162.1	142.1	200.1
**XLOC_014229**	STAT4	signal transducer and activator of transcription 4	0.6	1.2	0.4	1.4	0.8	1.4
**XLOC_014255**	SATB2	SATB homeobox 2	0.7	0.6	0.6	0.9	0.4	0.9
**XLOC_014288**	HDAC11	histone deacetylase 11	3.9	6.2	4.7	6.4	9.4	6.7
**XLOC_014356**	PAX7	paired box 7	0.1	0.0	0.0	0.0	0.0	0.0

**XLOC_014403**	DDX58	DEAD (Asp-Glu-Ala-Asp) box polypeptide 58	5.5	5.1	4.1	7.1	4.3	6.5
**XLOC_014460**	PRDM2	"PR domain containing 2, with ZNF domain"	19.7	11.3	23.3	10.3	30.9	10.7
**XLOC_014461**	LOC106040219	PR domain zinc finger protein 2-like	9.8	9.3	18.6	11.6	11.8	9.7
**XLOC_014471**	TNFRSF8	"tumor necrosis factor receptor superfamily, member 8"	0.6	0.8	0.6	0.8	1.5	0.8
**XLOC_014513**	FOXL2	forkhead box L2	439.3	342.7	489.9	372.4	336.8	369.7
**XLOC_014567**	LOC106040339	transcription cofactor HES-6-like	1.1	0.3	0.7	0.2	0.6	0.1
**XLOC_014681**	KLF4	Kruppel-like factor 4 (gut)	4.0	4.7	5.7	3.9	9.7	4.5
**XLOC_014741**	LOC106040490	mitogen-activated protein kinase 11-like	90.8	41.1	97.3	30.4	67.8	25.7
**XLOC_014834**	WNT5A	"wingless-type MMTV integration site family, member 5A"	5.0	11.0	2.5	9.9	2.8	9.7
**XLOC_014839**	HESX1	HESX homeobox 1	4.3	0.4	2.2	0.4	2.2	0.6
**XLOC_014849**	PKNOX2	PBXknotted 1 homeobox 2	32.9	18.4	15.0	22.2	7.4	24.3
**XLOC_014859**	FLI1	"Fli-1 proto-oncogene, ETS transcription factor"	5.1	17.9	2.0	19.2	4.3	18.5
**XLOC_014992**	LHX2	LIM homeobox 2	9.6	3.2	8.1	3.2	5.4	2.3
**XLOC_015009**	PBX3	pre-B-cell leukemia homeobox 3	7.9	10.2	7.4	11.5	9.7	11.9
**XLOC_015040**	TRAF1	TNF receptor-associated factor 1	22.4	9.6	22.2	8.8	15.2	6.9
**XLOC_015048**	LHX6	LIM homeobox 6	2.5	8.0	2.5	8.0	1.8	8.7
**XLOC_015066**	NR5A1	"nuclear receptor subfamily 5, group A, member 1"	84.7	57.2	59.5	65.4	41.4	64.7
**XLOC_015071**	NR6A1	"nuclear receptor subfamily 6, group A, member 1"	4.0	2.9	3.3	2.8	1.4	1.7
**XLOC_015093**	ZGPAT	"zinc finger, CCCH-type with G patch domain"	14.0	10.0	14.0	10.6	7.8	9.7
**XLOC_015109**	MYT1	myelin transcription factor 1	0.8	0.1	0.5	0.1	0.1	0.1
**XLOC_015124**	TCFL5	transcription factor-like 5 (basic helix-loop-helix)	1.9	0.7	2.4	0.9	1.0	0.5
**XLOC_015137**	TAF4	"TAF4 RNA polymerase II, TATA box binding protein (TBP)-associated factor, 135kDa"	2.7	3.5	1.8	3.8	2.8	3.8
**XLOC_015247**	TEAD1	TEA domain family member 1 (SV40 transcriptional enhancer factor)	6.2	12.5	5.6	12.8	15.3	14.8
**XLOC_015293**	ELF2	E74-like factor 2 (ets domain transcription factor)	21.7	19.6	17.2	18.6	19.0	20.8
**XLOC_015329**	NAA15	"N(alpha)-acetyltransferase 15, NatA auxiliary subunit"	62.4	17.3	35.3	18.8	28.1	19.9
**XLOC_015331**	NOCT	nocturnin	18.8	12.4	24.2	13.4	17.4	12.0

**XLOC_015377**	ZEB2	zinc finger E-box binding homeobox 2	15.1	35.4	9.3	40.6	12.2	36.2
**XLOC_015388**	MLXIPL	MLX interacting protein-like	0.8	0.4	0.8	0.4	0.8	0.3
**XLOC_015426**	MLXIPL	MLX interacting protein-like	1.7	0.4	0.9	0.3	0.3	0.2
**XLOC_015441**	HSF5	heat shock transcription factor family member 5	0.5	0.1	0.5	0.1	0.3	0.1
**XLOC_015470**	GTF2IRD1	GTF2I repeat domain containing 1	1.6	3.8	1.5	3.6	1.6	3.6
**XLOC_015480**	LOC106041112	GS homeobox 1-like	0.1	0.0			0.1	0.0
**XLOC_015560**	CIITA	"class II, major histocompatibility complex, transactivator"	4.9	2.6	9.7	1.5	6.3	1.6
**XLOC_015588**	USP7	ubiquitin specific peptidase 7 (herpes virus-associated)	64.1	27.5	41.1	30.5	36.0	30.1
**XLOC_015662**	PHTF1	putative homeodomain transcription factor 1	6.5	7.4	5.4	7.6	7.5	7.7
**XLOC_015710**	ELK4	"ELK4, ETS-domain protein (SRF accessory protein 1)"	2.1	7.7	1.3	8.1	4.0	7.6
**XLOC_015732**	LOC106041382	bile acid receptor-like	1.2	0.5	1.0	0.9	0.6	0.4
**XLOC_015804**	SOX13	SRY (sex determining region Y)-box 13	0.1	0.8	0.0	0.9	0.1	1.0
**XLOC_015913**	CUX1	cut-like homeobox 1	19.7	19.8	12.0	37.4	12.7	26.8
**XLOC_015929**	MYBBP1A	MYB binding protein (P160) 1a	22.0	17.1	23.2	19.8	20.7	22.2
**XLOC_015941**	ALX1	ALX homeobox 1	0.1	2.9	0.1	5.7	0.3	4.0
**XLOC_015974**	ATF6	activating transcription factor 6	28.0	24.7	25.6	25.3	19.7	24.5
**XLOC_015978**	NOTCH2	notch 2	41.3	40.4	42.5	36.5	18.1	30.9
**XLOC_016018**	DDR2	discoidin domain receptor tyrosine kinase 2	1.5	7.3	1.6	7.4	1.3	8.7
**XLOC_016026**	MTA1	metastasis associated 1	33.0	35.3	28.3	31.8	20.6	30.8
**XLOC_016047**	PRRX1	paired related homeobox 1	7.6	4.1	4.1	5.5	1.7	4.5
**XLOC_016054**	LOC106041605	pre-B-cell leukemia transcription factor 1	7.8	23.7	8.1	30.1	9.7	27.0
**XLOC_016144**	E2F3	E2F transcription factor 3	1.5	2.1	1.8	2.5	2.8	3.1
**XLOC_016322**	UBP1	upstream binding protein 1 (LBP-1a)	10.8	33.2	17.3	40.7	34.9	40.4
**XLOC_016355**	MAPK10	mitogen-activated protein kinase 10	0.1	0.2	0.4	0.7	0.2	0.3
**XLOC_016433**	ZNF281	zinc finger protein 281	19.9	8.6	10.3	9.2	8.7	8.6
**XLOC_016472**	LHX9	LIM homeobox 9	19.0	11.6	13.5	20.2	11.5	13.6

**XLOC_016525**	ZNF277	zinc finger protein 277	53.3	17.5	27.0	17.4	36.7	19.0
**XLOC_016533**	FOXP2	forkhead box P2	2.5	5.2	3.4	4.5	3.6	5.3
**XLOC_016559**	FOXP2	forkhead box P2	2.8	0.1	0.9	0.2	0.2	0.1
**XLOC_016606**	JMY	"junction mediating and regulatory protein, p53 cofactor"	6.0	6.2	5.6	5.7	9.0	5.5
**XLOC_016708**	LOC106042201	ubiquitin carboxyl-terminal hydrolase CYLD-like	3.4	6.4	4.9	5.6	4.0	5.3
	FOXA2	forkhead box A2			0.1	0.0	0.0	0.0
**XLOC_016854**	KLF12	Kruppel-like factor 12	4.7	3.9	5.8	2.9	8.6	3.8
**XLOC_016919**	ARRB1	"arrestin, beta 1"	16.8	46.7	16.9	54.4	11.6	47.9
**XLOC_017098**	LHX4	LIM homeobox 4	0.3	0.4	0.5	0.1	0.1	0.1
**XLOC_017164**	MSL3	male-specific lethal 3 homolog (Drosophila)	125.5	105.2	96.1	113.3	110.2	117.2
**XLOC_017174**	TBL1X	transducin (beta)-like 1X-linked	25.8	24.6	25.7	25.5	23.1	28.7
**XLOC_017261**	ZNF438	zinc finger protein 438	1.7	3.8	2.1	3.8	4.0	4.3
**XLOC_017273**	MKX	mohawk homeobox	2.2	21.0	2.6	29.5	1.9	40.5
**XLOC_017284**	ZEB1	zinc finger E-box binding homeobox 1	2.4	9.0	1.9	8.7	3.8	9.5
**XLOC_017303**	RAB18	"RAB18, member RAS oncogene family"	142.8	91.2	111.7	92.5	143.9	101.6
**XLOC_017314**	TRPS1	trichorhinophalangeal syndrome I	0.8	2.4	1.1	2.6	2.6	2.3
**XLOC_017384**	CREBRF	CREB3 regulatory factor	4.2	6.1	4.0	5.3	5.8	4.7
**XLOC_017391**	MSX2	msh homeobox 2	19.6	2.0	21.0	1.2	8.4	1.9
**XLOC_017393**	BRD8	bromodomain containing 8	27.6	16.1	24.5	15.8	16.3	15.4
**XLOC_017504**	POU6F2	POU class 6 homeobox 2	24.3	6.2	16.4	4.4	17.4	7.2
**XLOC_017566**	DMRT3	doublesex and mab-3 related transcription factor 3	0.0	0.0	0.0	0.1	0.1	0.0
**XLOC_017568**	DMRT2	doublesex and mab-3 related transcription factor 2	4.3	1.0	4.0	0.5	3.0	0.6
**XLOC_017615**	TBX22	T-box 22	1.1	0.9	1.1	0.3	0.4	0.3
**XLOC_017667**	HDX	highly divergent homeobox	7.5	5.7	9.0	5.7	9.1	5.3
**XLOC_017760**	LOC106043073	iroquois-class homeodomain protein irx-3-like	34.3	5.7	9.2	11.9	2.1	9.9
**XLOC_017801**	FOXN2	forkhead box N2	9.6	8.4	5.1	9.4	6.7	9.8

**XLOC_017804**	GTF2A1L	"general transcription factor IIA, 1-like"	10.8	1.4	3.7	1.6	1.8	0.9
**XLOC_017818**	GTF2A1L	"general transcription factor IIA, 1-like"	0.1	0.2	0.0	0.1	0.1	0.2
**XLOC_017862**	LOC106043168	homeobox protein not2-like	3.9	0.1	1.7	0.2	0.8	0.2
**XLOC_017874**	VAX2	ventral anterior homeobox 2	0.2	0.7	0.2	0.8	0.2	0.9
**XLOC_017907**	SMAD9	SMAD family member 9	29.9	27.6	24.9	33.6	25.4	38.4
**XLOC_017928**	SMAD9	SMAD family member 9	1.4	4.7	2.2	4.0	4.7	4.5
**XLOC_017990**	NR0B1	"nuclear receptor subfamily 0, group B, member 1"	59.2	142.2	49.3	139.9	71.1	125.1
**XLOC_017992**	TAB3	TGF-beta activated kinase 1MAP3K7 binding protein 3	13.0	11.1	10.4	13.0	12.9	13.3
**XLOC_018048**	ARNT2	aryl-hydrocarbon receptor nuclear translocator 2	14.9	11.0	8.0	10.9	6.7	10.3
**XLOC_018058**	BNC1	basonuclin 1	4.8	1.7	4.5	2.5	5.8	1.7
**XLOC_018101**	RUNX1	runt-related transcription factor 1	1.8	7.3	1.6	8.2	4.8	7.1
**XLOC_018119**	PAXBP1	PAX3 and PAX7 binding protein 1	25.9	24.1	20.8	24.7	29.8	24.4
**XLOC_018160**	BACH1	"BTB and CNC homology 1, basic leucine zipper transcription factor 1"	14.1	14.8	13.4	15.3	10.8	13.2
**XLOC_018202**	MAFB	v-maf avian musculoaponeurotic fibrosarcoma oncogene homolog B	23.6	64.1	7.7	49.9	12.3	40.9
**XLOC_018203**	ZHX3	zinc fingers and homeoboxes 3	1.5	4.2	1.3	5.2	1.1	3.8
**XLOC_018329**	LHX8	LIM homeobox 8	27.9	12.0	38.5	11.3	37.5	7.9
**XLOC_018439**	SMAD3	SMAD family member 3	25.8	31.4	19.3	36.8	25.2	30.5
**XLOC_018440**	SMAD6	SMAD family member 6	7.6	13.5	7.8	11.4	7.9	12.2
**XLOC_018590**	NF1	neurofibromin 1	7.6	8.3	4.9	8.2	8.1	8.2
**XLOC_018632**	NF1	neurofibromin 1	8.1	21.3	23.7	18.0	103.3	17.3
**XLOC_018633**	NF1	neurofibromin 1	79.7	17.3	23.8	20.3	50.0	12.1
**XLOC_018648**	LHX1	LIM homeobox 1	1.2	0.3	1.5	0.1	0.8	0.3
**XLOC_018651**	PSIP1	PC4 and SFRS1 interacting protein 1	160.7	59.5	131.4	53.7	188.8	72.7
**XLOC_018657**	NFIB	nuclear factor IB	6.5	8.1	3.9	8.0	5.1	9.0
**XLOC_018682**	RIPK1	receptor (TNFRSF)-interacting serine-threonine kinase 1	31.7	17.1	21.4	20.7	21.6	21.5
**XLOC_018697**	LOC106043843	forkhead box protein F2-like	0.0	0.0	0.0	0.1	0.0	0.0

**XLOC_018699**	IRF4	interferon regulatory factor 4	1.1	1.3	2.2	0.8	1.3	0.9
**XLOC_018729**	NR4A2	"nuclear receptor subfamily 4, group A, member 2"	1.0	0.5	1.5	0.5	0.7	0.5
**XLOC_018767**	XBP1	X-box binding protein 1	132.5	234.1	175.8	232.3	170.0	262.5
**XLOC_018853**	NAAA	N-acylethanolamine acid amidase	166.3	34.8	42.6	57.3	22.7	52.0
**XLOC_018912**	OTX2	orthodenticle homeobox 2	43.1	3.3	20.2	3.2	8.3	2.1
**XLOC_018914**	TBPL2	TATA box binding protein like 2	29.6	5.3	18.6	7.1	9.0	3.6
**XLOC_018992**	GTF3C4	"general transcription factor IIIC, polypeptide 4, 90kDa"	32.3	11.7	21.7	9.6	18.8	9.3
**XLOC_019050**	REXO4	"REX4 homolog, 3'-5' exonuclease"	328.3	80.0	153.7	85.5	72.5	89.3
	RORB	RAR-related orphan receptor B			0.0	0.0	0.0	0.0
**XLOC_019252**	PEX14	peroxisomal biogenesis factor 14	7.7	9.1	6.7	9.0	8.0	8.9
**XLOC_019272**	RERE	arginine-glutamic acid dipeptide (RE) repeats	86.6	12.2	16.4	14.3	8.4	10.8
**XLOC_019313**	SFRP4	secreted frizzled-related protein 4	7.4	65.6	8.3	86.3	11.3	72.7
**XLOC_019332**	KAT6A	K(lysine) acetyltransferase 6A	15.5	13.3	10.7	12.8	9.2	12.9
**XLOC_019334**	NKX6-3	NK6 homeobox 3	157.2	14.2	70.9	32.2	11.5	9.6
**XLOC_019366**	LZTS1	"leucine zipper, putative tumor suppressor 1"	0.1	0.8	0.1	0.5	1.4	0.7
**XLOC_019370**	IKBKB	"inhibitor of kappa light polypeptide gene enhancer in B-cells, kinase beta"	77.3	33.8	55.2	35.2	42.0	37.0
**XLOC_019405**	LZTS1	"leucine zipper, putative tumor suppressor 1"	35.8	24.3	19.9	18.1	10.0	19.5
**XLOC_019485**	GCFC2	GC-rich sequence DNA-binding factor 2	23.3	9.9	18.0	9.3	16.4	8.9
**XLOC_019547**	GTF2A1	"general transcription factor IIA, 1, 1937kDa"	9.1	11.8	8.8	13.2	9.5	15.1
**XLOC_019618**	LOC106044620	transcription factor HES-5-like	0.8	0.9	1.0	0.6	1.4	0.6
**XLOC_019656**	LOC106044624	transcription factor HES-5-like	0.1	0.1	0.3	0.1	0.1	0.1
**XLOC_019657**	LOC106044623	transcription factor HES-5-like	0.0	0.0	0.0	0.0	0.1	0.1
**XLOC_019729**	MEIS1	Meis homeobox 1	14.8	28.8	7.1	33.1	9.0	26.7
**XLOC_019745**	VSX1	visual system homeobox 1	1.0	0.2	0.3	0.5	0.2	0.3
**XLOC_019828**	TP63	tumor protein p63	16.9	5.8	19.1	6.3	10.0	3.6
**XLOC_019884**	LOC106044855	hepatocyte nuclear factor 4-beta-like	3.4	3.9	5.1	3.9	4.4	4.0

**XLOC_019906**	LOC106044841	forkhead box protein C2-like	2.7	1.9	2.6	0.9	0.7	1.0
**XLOC_019940**	TCF4	transcription factor 4	0.5	1.1	0.3	1.5	0.6	1.4
**XLOC_019951**	MALT1	MALT1 paracaspase	11.6	10.0	14.5	9.7	12.7	8.4
**XLOC_019965**	TCF4	transcription factor 4	9.0	21.8	5.4	24.1	5.4	23.1
**XLOC_019974**	RAX	retina and anterior neural fold homeobox	0.0	0.0	0.0	0.1	0.0	0.1
**XLOC_020021**	CERS4	ceramide synthase 4	14.7	16.1	19.3	11.5	13.7	14.5
**XLOC_020048**	UHRF1	ubiquitin-like with PHD and ring finger domains 1	49.2	17.7	36.9	19.3	36.8	32.1
**XLOC_020059**	CREB3L3	cAMP responsive element binding protein 3-like 3	0.3	0.2	0.0	0.3	0.1	0.3
**XLOC_020114**	ZXDC	ZXD family zinc finger C	4.2	5.1	5.0	5.8	4.2	4.6
**XLOC_020117**	KLF15	Kruppel-like factor 15	14.8	11.5	20.4	9.7	9.9	7.1
**XLOC_020181**	NFIL3	"nuclear factor, interleukin 3 regulated"	13.9	17.0	10.9	15.6	10.1	13.4
**XLOC_020189**	TAF12	"TAF12 RNA polymerase II, TATA box binding protein (TBP)-associated factor, 20kDa"	23.2	30.2	31.1	32.0	32.6	37.0
**XLOC_020214**	LOC106045119	transcription factor E2F2-like	1.4	1.6	0.5	1.3	0.5	2.1
**XLOC_020215**	LOC106045127	DNA-binding protein inhibitor ID-3-A-like	142.6	378.6	130.9	644.3	72.4	543.2
**XLOC_020218**	TAF12	"TAF12 RNA polymerase II, TATA box binding protein (TBP)-associated factor, 20kDa"	32.8	2.0	6.7	0.8	0.0	0.4
**XLOC_020309**	VAX1	ventral anterior homeobox 1	2.8	1.0	2.9	0.7	1.2	0.2
**XLOC_020337**	EMX2	empty spiracles homeobox 2	38.6	24.4	38.6	20.1	21.6	18.5
**XLOC_020382**	SALL1	spalt-like transcription factor 1	12.9	15.3	13.2	11.8	11.5	11.5
**XLOC_020418**	CREM	cAMP responsive element modulator	10.3	16.5	12.9	18.2	18.2	16.0
**XLOC_020422**	NFKB1	nuclear factor of kappa light polypeptide gene enhancer in B-cells 1	49.8	35.3	24.7	43.0	19.4	37.2
**XLOC_020537**	TGIF2	TGFB-induced factor homeobox 2	3.5	8.9	3.0	10.5	3.2	10.9
**XLOC_020540**	L3MBTL1	l(3)mbt-like 1 (Drosophila)	0.3	0.6	0.3	0.5	0.2	0.4
**XLOC_020563**	NCOA6	nuclear receptor coactivator 6	11.0	8.3	10.0	7.9	9.8	8.5
**XLOC_020566**	TGIF2	TGFB-induced factor homeobox 2	0.8	1.6	0.7	1.6	0.9	1.5
**XLOC_020567**	TGIF2	TGFB-induced factor homeobox 2	17.0	13.0	7.1	13.1	11.3	12.8
**XLOC_020574**	ITCH	itchy E3 ubiquitin protein ligase	4.4	7.8	4.4	7.5	9.5	8.2

**XLOC_020584**	TLE4	transducin-like enhancer of split 4	3.6	6.4	4.4	8.3	8.1	8.3
**XLOC_020593**	TLE1	"transducin-like enhancer of split 1 (E(sp1) homolog, Drosophila)"	9.8	12.5	8.6	14.4	11.3	15.7
**XLOC_020605**	NOBOX	NOBOX oogenesis homeobox	8.8	1.9	6.6	2.4	2.4	1.0
**XLOC_020674**	EDA2R	ectodysplasin A2 receptor	8.8	17.9	12.8	18.8	13.4	17.4
**XLOC_020675**	LOC106045553	heat shock factor protein 3-like	25.3	28.6	31.9	27.2	29.3	26.5
**XLOC_020701**	AR	androgen receptor	19.3	29.9	19.7	42.5	23.9	35.3
**XLOC_020737**	OTX1	orthodenticle homeobox 1	0.5	0.5	0.6	0.1	0.0	0.5
**XLOC_020785**	LOC106045650	nuclear receptor ROR-beta-like	2.0	0.3	2.0	0.2	0.4	0.2
**XLOC_020816**	LOC106045683	mucosa-associated lymphoid tissue lymphoma translocation protein 1-like	0.8	1.2	0.4	1.9	0.2	1.4
**XLOC_020819**	HMG20B	high mobility group 20B	177.2	148.1	221.7	159.2	103.9	146.5
**XLOC_020823**	NFIC	nuclear factor IC (CCAAT-binding transcription factor)	16.1	80.5	14.5	81.3	15.9	76.6
**XLOC_020829**	TCF3	transcription factor 3	11.3	17.0	9.5	19.4	7.0	19.9
**XLOC_020847**	TBX15	T-box 15	0.0	0.1	0.0	0.0	0.0	0.0
**XLOC_020867**	LOC106045720	glycogen synthase kinase-3 beta-like	205.2	37.9	57.0	61.4	37.3	60.9
**XLOC_020963**	TFEB	transcription factor EB	1.2	4.0	1.7	4.6	0.7	4.3
**XLOC_020993**	BTG2	"BTG family, member 2"	63.2	73.6	77.3	114.9	66.2	62.9
**XLOC_021002**	MDFI	MyoD family inhibitor	1.3	18.7	0.7	27.3	0.4	20.3
**XLOC_021005**	FOXP4	forkhead box P4	5.9	9.2	7.9	8.7	7.1	9.4
**XLOC_021020**	LZTR1	leucine-zipper-like transcription regulator 1	34.5	18.4	39.6	15.8	32.3	15.7
**XLOC_021257**	HOPX	HOP homeobox	0.4	1.2	0.2	1.1	0.7	1.3
**XLOC_021259**	REST	RE1-silencing transcription factor	8.0	13.7	5.7	14.4	19.5	17.0
**XLOC_021313**	PPARD	peroxisome proliferator-activated receptor delta	9.4	14.7	11.9	15.9	7.9	14.6
**XLOC_021324**	MAPK14	mitogen-activated protein kinase 14	119.7	67.5	138.1	64.8	109.7	62.4
**XLOC_021332**	ELF3	"E74-like factor 3 (ets domain transcription factor, epithelial-specific)"	6.4	5.1	7.3	13.8	2.5	13.1
**XLOC_021343**	NFYA	"nuclear transcription factor Y, alpha"	10.9	15.9	14.1	15.5	22.0	15.8
**XLOC_021484**	MXD1	MAX dimerization protein 1	16.4	10.1	11.8	12.1	6.6	10.1

**XLOC_021556**	UBN1	ubinuclein 1	10.4	6.2	7.1	6.1	6.3	5.3
**XLOC_021681**	TFDP1	transcription factor Dp-1	6.1	12.7	6.0	16.3	10.6	22.7
**XLOC_021707**	NFX1	"nuclear transcription factor, X-box binding 1"	16.6	14.2	27.0	12.4	31.1	11.8
**XLOC_021744**	ZFPM1	"zinc finger protein, FOG family member 1"	64.5	36.5	53.2	31.0	28.0	34.4
**XLOC_021772**	CBFA2T3	"core-binding factor, runt domain, alpha subunit 2%3B translocated to, 3"	0.3	2.4	0.2	2.4	0.1	2.4
**XLOC_021843**	SCMH1	sex comb on midleg homolog 1 (Drosophila)	19.6	11.4	13.0	12.4	7.1	11.1
**XLOC_021857**	NFAT5	"nuclear factor of activated T-cells 5, tonicity-responsive"	4.3	9.1	3.3	7.9	6.8	7.9
**XLOC_021858**	WWP2	WW domain containing E3 ubiquitin protein ligase 2	19.3	13.1	13.5	14.3	6.5	13.1
**XLOC_021863**	ZFHX3	zinc finger homeobox 3	1.5	2.6	1.9	2.7	1.1	2.3
**XLOC_021893**	TERF2IP	"telomeric repeat binding factor 2, interacting protein"	12.7	8.6	10.2	8.1	13.6	9.7
**XLOC_021897**	NOD1	nucleotide-binding oligomerization domain containing 1	1.4	3.5	1.4	4.0	2.8	3.4
	TBX4	T-box 4					0.0	0.0
**XLOC_022151**	TBX2	T-box 2	1.0	13.6	0.1	12.9	0.3	14.6
**XLOC_022223**	PSMC5	"proteasome (prosome, macropain) 26S subunit, ATPase, 5"	114.5	133.4	111.5	131.5	89.7	160.8
**XLOC_022326**	TRAF3	TNF receptor-associated factor 3	6.2	2.0	4.3	2.5	2.8	1.7
**XLOC_022328**	RCOR1	REST corepressor 1	5.6	11.1	6.0	13.3	17.3	13.8
**XLOC_022351**	IGHMBP2	immunoglobulin mu binding protein 2	0.5	0.2	0.1	1.2	0.0	0.2
**XLOC_022386**	SIGIRR	single immunoglobulin and toll-interleukin 1 receptor (TIR) domain	0.8	0.9	1.5	0.8	0.7	0.7
**XLOC_022388**	IGHMBP2	immunoglobulin mu binding protein 2	10.1	13.4	17.1	12.7	15.3	11.4
**XLOC_022455**	FOXK2	forkhead box K2	57.8	26.6	34.6	30.7	25.1	30.1
**XLOC_022508**	LOC106047120	interferon regulatory factor 4-like	1.1	2.1	0.0	1.5	0.4	3.0
**XLOC_022512**	PLAGL2	pleiomorphic adenoma gene-like 2	1.9	2.1	2.5	2.0	2.2	2.0
**XLOC_022544**	MYOCD	myocardin	2.3	6.7	2.6	5.6	3.8	5.8
**XLOC_022650**	PRRX2	paired related homeobox 2	0.4	1.2	0.1	1.2	0.1	1.1
**XLOC_022677**	NKX6-2	NK6 homeobox 2	34.0	2.1	10.3	4.9	3.0	3.7
**XLOC_022850**	LOC106047451	mucosa-associated lymphoid tissue lymphoma translocation protein 1-like	23.9	14.0	12.6	16.5	12.1	16.9

**XLOC_022925**	LOC106047487	brain-specific homeoboxPOU domain protein 3	12.2	1.8	5.5	1.0	1.8	0.8
**XLOC_022959**	NPAT	"nuclear protein, ataxia-telangiectasia locus"	10.5	6.7	8.2	7.5	7.0	6.3
**XLOC_022966**	MEF2C	myocyte enhancer factor 2C	18.3	11.6	9.4	10.5	13.1	11.3
**XLOC_023103**	TSC22D2	"TSC22 domain family, member 2"	29.2	22.4	27.8	25.7	19.8	24.4
**XLOC_023104**	LOC106047670	TSC22 domain family protein 2-like	101.4	80.2	87.8	99.0	39.8	120.1
**XLOC_023126**	DIP2B	disco-interacting protein 2 homolog B	10.9	12.3	13.6	10.9	16.2	11.2
**XLOC_023127**	ATF1	activating transcription factor 1	16.1	46.5	26.5	49.9	26.4	55.1
**XLOC_023136**	ATG101	autophagy related 101	79.7	71.6	96.1	69.3	65.2	72.1
**XLOC_023175**	TOX2	TOX high mobility group box family member 2	22.9	15.4	24.8	10.4	30.0	13.9
**XLOC_023226**	PBX1	pre-B-cell leukemia homeobox 1	67.3	67.9	23.3	86.5	10.4	79.7
**XLOC_023229**	ZNF648	zinc finger protein 648	22.6	27.3	25.6	29.8	16.0	30.8
**XLOC_023282**	CHD4	chromodomain helicase DNA binding protein 4	125.5	56.3	129.2	56.8	77.4	45.2
**XLOC_023370**	NR0B2	"nuclear receptor subfamily 0, group B, member 2"	5.2	5.5	6.7	4.1	12.3	4.6
**XLOC_023395**	RFXANK	regulatory factor X-associated ankyrin-containing protein	7.8	17.7	9.4	19.3	7.4	18.0
**XLOC_023399**	GATAD2A	GATA zinc finger domain containing 2A	8.1	6.9	4.6	8.3	4.0	7.6
**XLOC_023481**	LOC106047988	uncharacterized LOC106047988	6.5	11.5	7.0	14.2	6.0	14.2
**XLOC_023486**	LOC106047991	signal transducer and activator of transcription 5A-like	13.4	26.4	11.6	32.1	10.8	27.5
**XLOC_023487**	STAT3	signal transducer and activator of transcription 3 (acute-phase response factor)	44.3	78.4	41.0	109.1	20.8	84.0
	PHOX2B	paired-like homeobox 2b			0.0	0.2		
**XLOC_023547**	LOC106048073	homeobox protein NANOG-like	0.0	0.0	0.0	0.0	0.0	0.0
**XLOC_023562**	FOXJ2	forkhead box J2	34.5	26.5	22.0	27.3	16.9	27.8
**XLOC_023563**	LOC106048065	homeobox protein NANOG-like	23.7	7.0	25.3	8.8	15.0	3.7
**XLOC_023605**	FOXM1	forkhead box M1	28.9	9.5	33.6	9.8	23.2	10.1
**XLOC_023726**	TRAPPC2	trafficking protein particle complex 2	92.7	152.7	164.8	176.2	225.6	160.2
**XLOC_023780**	MYRF	myelin regulatory factor	8.6	17.9	6.9	17.2	6.3	20.9
**XLOC_023817**	SNF8	"SNF8, ESCRT-II complex subunit"	61.6	45.8	77.5	47.4	58.1	57.9

**XLOC_023823**	HOXB9	homeobox B9	4.4	5.0	1.5	5.6	1.4	5.9
**XLOC_023824**	HOXB8	homeobox B8	0.4	0.6	0.2	1.0	0.4	0.4
**XLOC_023825**	HOXB7	homeobox B7	5.7	8.4	2.0	8.1	4.0	8.9
**XLOC_023826**	HOXB6	homeobox B6	4.4	14.0	2.7	13.0	3.2	13.1
**XLOC_023832**	HOXB4	homeobox B4	11.1	22.0	5.6	23.9	3.6	21.5
**XLOC_023834**	HOXB3	homeobox B3	1.3	2.7	0.7	2.4	0.7	2.3
**XLOC_023835**	HOXB2	homeobox B2	1.1	1.4	0.6	1.2	0.1	1.0
**XLOC_023847**	NFE2L1	"nuclear factor, erythroid 2-like 1"	24.7	28.2	22.5	29.2	14.9	28.0
**XLOC_023921**	LOC106048406	nuclear factor interleukin-3-regulated protein	0.0	0.0	0.0	0.1	0.0	0.0
**XLOC_024145**	LOC106048608	zinc finger and SCAN domain-containing protein 29	4.0	1.1	1.7	1.3	1.1	1.2
**XLOC_024238**	POU6F1	POU class 6 homeobox 1	0.5	4.5	0.2	3.1	0.3	4.1
**XLOC_024239**	TFCP2	transcription factor CP2	53.5	51.2	45.1	59.2	33.5	53.0
**XLOC_024240**	CSRNP2	cysteine-serine-rich nuclear protein 2	7.7	11.6	5.1	12.7	3.8	10.0
**XLOC_024345**	SOST	sclerostin	3.6	1.0	3.0	0.6	1.8	0.7
**XLOC_024346**	MEOX1	mesenchyme homeobox 1	0.2	0.2	0.0	0.4	0.0	0.3
**XLOC_024347**	ETV4	ets variant 4	3.1	2.2	3.7	2.2	1.3	1.3
**XLOC_024360**	LOC106048786	ras-related protein Rab-18-B-like	20.9	14.0	11.5	21.8	14.8	22.7
**XLOC_024364**	ETV4	ets variant 4	5.2	0.0	0.5	0.1	0.0	0.1
**XLOC_024377**	TAF5L	"TAF5-like RNA polymerase II, p300CBP-associated factor (PCAF)-associated factor, 65kDa"	21.4	14.2	19.5	16.4	10.7	13.7
**XLOC_024389**	LOC106048825	iroquois-class homeodomain protein IRX-4-like	1.7	1.5	3.3	1.5	2.1	0.7
**XLOC_024448**	ATF7	activating transcription factor 7	1.4	3.4	1.7	3.7	1.3	4.1
**XLOC_024453**	HOXC4	homeobox C4	0.9	1.0	0.2	0.8	0.3	0.8
**XLOC_024454**	HOXC5	homeobox C5	0.7	4.1	0.4	2.7	0.4	2.4
**XLOC_024456**	LOC106048882	homeobox protein Hox-C6	8.1	38.2	5.6	36.2	5.6	31.0
**XLOC_024460**	HOXC8	homeobox C8	2.6	9.9	0.4	11.4	1.5	9.5
**XLOC_024461**	HOXC9	homeobox C9	1.6	4.6	0.4	5.9	0.8	7.2

**XLOC_024462**	HOXC10	homeobox C10	0.2	0.1	0.0	0.1	0.3	1.2
	HOXC11	homeobox C11			0.0	0.0		
**XLOC_024465**	HOXC13	homeobox C13	0.5	0.0	0.6	0.0	0.2	0.0
**XLOC_024513**	IRF5	interferon regulatory factor 5	4.2	5.5	5.4	4.3	4.4	4.4
**XLOC_024521**	NRF1	nuclear respiratory factor 1	11.9	12.6	8.5	9.6	7.1	7.6
**XLOC_024564**	LOC106048963	myocyte-specific enhancer factor 2D-like	2.3	9.6	4.1	10.9	2.9	11.0
**XLOC_024613**	NAB2	NGFI-A binding protein 2 (EGR1 binding protein 2)	2.4	4.5	1.0	4.5	0.8	4.9
**XLOC_024643**	STAT6	"signal transducer and activator of transcription 6, interleukin-4 induced"	13.4	28.6	18.1	27.5	14.6	26.5
**XLOC_024654**	SMARCD3	"SWISNF related, matrix associated, actin dependent regulator of chromatin, subfamily d, member 3"	9.7	13.5	14.6	12.8	17.6	12.9
**XLOC_024731**	NPAS2	neuronal PAS domain protein 2	1.0	3.0	0.9	3.3	0.9	3.5
**XLOC_024769**	GATAD2B	GATA zinc finger domain containing 2B	27.4	23.7	28.9	23.7	21.2	23.3
**XLOC_024839**	FOXN1	forkhead box N1	1.9	0.3	1.7	0.2	1.0	0.1
**XLOC_024925**	IRF8	interferon regulatory factor 8	22.8	27.9	28.8	32.7	16.4	27.8
**XLOC_024940**	CERS2	ceramide synthase 2	58.6	55.2	69.0	51.5	55.3	51.5
**XLOC_024942**	ARNT	aryl hydrocarbon receptor nuclear translocator	24.0	37.8	32.5	36.0	30.0	35.1
**XLOC_025013**	LOC106049319	E3 ubiquitin-protein ligase RNF19B-like	3.5	9.8	3.4	9.7	4.2	8.0
**XLOC_025069**	SCRT2	scratch family zinc finger 2	0.7	0.2	0.4	0.4	0.0	0.1
**XLOC_025086**	LOC106049386	general transcription factor IIH subunit 2	16.2	8.6	14.1	8.8	12.1	10.1
**XLOC_025180**	HSF1	heat shock transcription factor 1	59.5	51.3	108.5	60.8	82.1	61.8
**XLOC_025192**	LOC106049483	LIM homeobox transcription factor 1-alpha-like	3.3	3.2	2.7	2.9	2.0	3.0
**XLOC_025204**	PBX4	pre-B-cell leukemia homeobox 4	28.0	14.8	18.8	19.7	12.1	19.2
**XLOC_025224**	LOC106049496	PR domain zinc finger protein 5-like	2.7	3.4	1.7	4.3	1.5	3.9
**XLOC_025255**	LOC106049528	uncharacterized LOC106049528	19.7	79.3	24.0	74.6	20.3	55.3
**XLOC_025262**	HDAC8	histone deacetylase 8	19.1	25.9	28.9	22.9	24.8	26.4
**XLOC_025315**	LOC106049581	transcription factor Sp1-like	9.0	17.6	6.2	17.8	7.5	21.2
**XLOC_025317**	VDR	"vitamin D (1,25- dihydroxyvitamin D3) receptor"	9.4	3.3	9.0	4.6	3.6	3.6

**XLOC_025336**	ISL1	ISL LIM homeobox 1	0.3	0.1	0.3	0.0	0.4	0.0
**XLOC_025360**	LOC106049620	platelet glycoprotein VI-like	0.4	0.5	0.3	0.2	0.3	0.2
**XLOC_025452**	LOC106049689	uncharacterized LOC106049689	1.6	0.2	0.5	0.2	0.1	0.1
**XLOC_025499**	KAT7	K(lysine) acetyltransferase 7	34.7	25.9	40.7	22.8	42.1	24.5
	LOC106049761	E3 ubiquitin-protein ligase TRIM39-like			0.1	0.0	0.0	0.0
**XLOC_025525**	LOC106049750	zinc finger protein 883-like	4.4	7.1	4.8	7.1	4.5	6.6
**XLOC_025546**	TP53BP1	tumor protein p53 binding protein 1	408.2	34.4	81.1	40.6	61.2	41.8
**XLOC_025550**	DNMT1	DNA (cytosine-5-)-methyltransferase 1	14.0	24.9	11.3	29.7	10.9	29.6
**XLOC_025729**	GTF3C2	"general transcription factor IIIC, polypeptide 2, beta 110kDa"	18.5	18.9	20.6	18.0	14.4	18.6
**XLOC_025887**	LOC106029359	uncharacterized LOC106029359	4.5	11.9	4.6	9.3	5.0	10.3
**XLOC_025888**	PA2G4	"proliferation-associated 2G4, 38kDa"	118.4	83.0	123.7	77.8	103.1	102.5
**XLOC_026016**	TAF6	"TAF6 RNA polymerase II, TATA box binding protein (TBP)-associated factor, 80kDa"	11.4	21.0	14.5	19.4	10.5	22.9
**XLOC_026083**	LOC106029471	transcription activator BRG1	121.3	76.0	134.3	80.4	72.7	77.8
**XLOC_026149**	LOC106029507	proto-oncogene c-Fos-like	20.0	2.5	9.0	3.2	1.1	1.7
**XLOC_026439**	LOC106029618	chromobox protein homolog 5-like	186.6	81.9	73.1	108.8	49.1	152.6

Table S8 Analysis of autophagy-related genes during the period between egg-laying and broody goose follicles

**gene_id**			**LSWF**	**BSWF**	**LLWF**	**BLWF**	**LSYF**	**BSYF**
**XLOC_002484**	MAP1LC3C	microtubule-associated protein 1 light chain 3 gamma	9.6	6.9	16.4	5.6	18.4	6.3
**XLOC_003943**	DRAM1	DNA-damage regulated autophagy modulator 1	9.2	15.4	12.3	26.3	28.6	17.8
**XLOC_004075**	DRAM1	DNA-damage regulated autophagy modulator 1	5.4	2.1	5.2	2.1	5.6	2.6
**XLOC_004173**	ULK1	unc-51 like autophagy activating kinase 1	6.4	7.8	10.9	7.5	14.5	6.4
**XLOC_004463**	LOC106031353	"dynein light chain 1, cytoplasmic-like"	1.7	2.5	1.3	2.8	1.4	3.8
**XLOC_005977**	SQSTM1	sequestosome 1	200.9	162.6	260.0	140.4	205.5	133.1
**XLOC_007973**	WIPI1	"WD repeat domain, phosphoinositide interacting 1"	44.2	26.6	39.7	27.5	25.2	25.0
**XLOC_008698**	MTDH	metadherin	40.0	45.6	34.4	51.6	42.0	57.9
**XLOC_008801**	TMEM74	transmembrane protein 74	0.3	0.3	0.3	0.8	0.4	0.4
**XLOC_009913**	PRKAA1	"protein kinase, AMP-activated, alpha 1 catalytic subunit"	28.4	16.5	32.9	20.7	26.2	16.7
**XLOC_010234**	DAP	death-associated protein	96.9	153.0	144.3	168.6	407.5	195.6
**XLOC_011156**	EPG5	ectopic P-granules autophagy protein 5 homolog (C. elegans)	14.4	9.5	16.3	8.3	18.0	9.2
**XLOC_012439**	RB1CC1	RB1-inducible coiled-coil 1	60.0	23.5	46.0	23.9	43.9	20.8
**XLOC_012611**	ATG4A	"autophagy related 4A, cysteine peptidase"	20.2	34.7	20.9	31.0	25.0	30.8
**XLOC_014275**	ATG7	autophagy related 7	5.9	6.9	6.3	6.7	6.4	7.3
**XLOC_014516**	PIK3CB	"phosphatidylinositol-4,5-bisphosphate 3-kinase, catalytic subunit beta"	22.5	6.8	7.0	8.7	5.6	8.9
**XLOC_014592**	ATG16L1	autophagy related 16-like 1	85.5	56.6	83.8	57.8	55.4	56.8
**XLOC_015157**	RGS19	regulator of G-protein signaling 19	26.0	61.0	13.5	77.2	10.4	69.4
**XLOC_016515**	LRRK2	leucine-rich repeat kinase 2	1.9	5.2	1.8	5.3	4.0	5.8
**XLOC_016948**	UVRAG	UV radiation resistance associated	120.3	49.3	136.7	48.8	95.1	35.5
**XLOC_017352**	ATG10	autophagy related 10	9.1	6.7	11.3	7.6	10.6	8.6

**XLOC_018915**	ATG14	autophagy related 14	4.4	4.0	5.1	4.2	4.1	3.3
**XLOC_018981**	ABL1	"ABL proto-oncogene 1, non-receptor tyrosine kinase"	20.9	35.5	19.7	37.1	15.4	34.4
**XLOC_019486**	EVA1A	eva-1 homolog A (C. elegans)	1.0	1.7	0.4	1.9	0.2	1.3
**XLOC_019810**	HTR2B	"5-hydroxytryptamine (serotonin) receptor 2B, G protein-coupled"	1.7	3.6	0.9	4.1	1.0	2.4
**XLOC_020423**	CISD2	CDGSH iron sulfur domain 2	41.6	48.8	66.5	45.0	96.9	63.5
**XLOC_020924**	ULK2	unc-51 like autophagy activating kinase 2	11.6	20.4	14.9	17.7	21.1	17.8
**XLOC_021338**	LOC106046118	uncharacterized LOC106046118	0.6	2.0	1.3	1.6	0.8	1.8
**XLOC_021737**	MAP1LC3B	microtubule-associated protein 1 light chain 3 beta	77.9	75.9	66.8	75.0	54.6	90.8
**XLOC_022123**	VMP1	vacuole membrane protein 1	22.4	21.4	34.2	19.6	34.8	20.3
**XLOC_022428**	WDR45B	WD repeat domain 45B	36.5	38.8	60.1	37.3	79.8	41.6
**XLOC_022589**	PARK7	parkinson protein 7	128.3	118.7	160.9	116.0	166.9	135.3
**XLOC_023496**	BECN1	"beclin 1, autophagy related"	190.1	59.9	76.3	65.2	31.7	67.2

Figure S1 Map of gene expression density

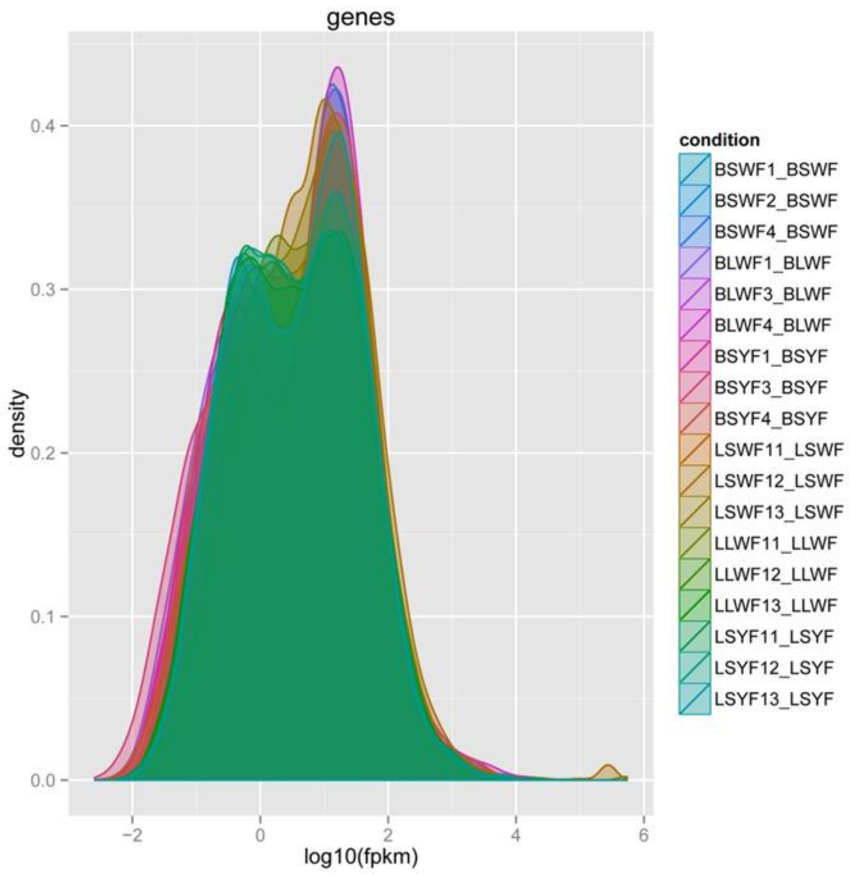

Origin picture of Figure 6c

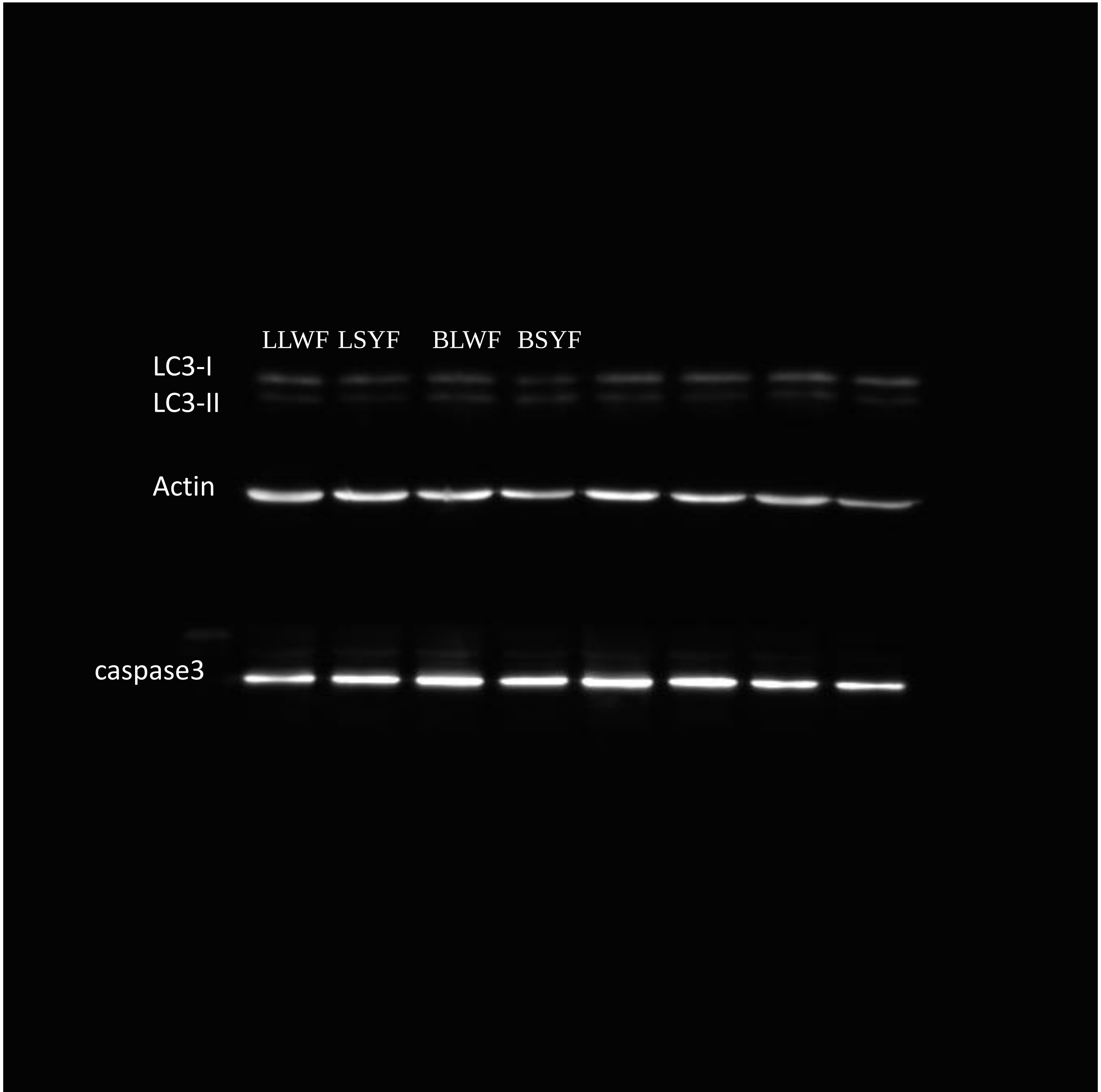

Supplement: Supplementary Information [file srep36877-s1.pdf]
